# Nrf2 is predominantly expressed in hippocampal neurons in a rat model of temporal lobe epilepsy

**DOI:** 10.1186/s13578-022-00951-y

**Published:** 2023-01-04

**Authors:** Sereen Sandouka, Aseel Saadi, Prince Kumar Singh, Rhoda Olowe, Tawfeeq Shekh-Ahmad

**Affiliations:** The Institute for Drug Research, The School of Pharmacy, Faculty of Medicine, The Hebrew University of Jerusalem, 91120 Jerusalem, Israel

**Keywords:** Nrf2, Oxidative stress, Antioxidants, Status epilepticus, Kainic Acid

## Abstract

**Background:**

Drug resistance is a particular problem in patients with temporal lobe epilepsy, where seizures originate mainly from the hippocampus. Many of these epilepsies are acquired conditions following an insult to the brain such as a prolonged seizure. Such conditions are characterized by pathophysiological mechanisms including massive oxidative stress that synergistically mediate the secondary brain damage, contributing to the development of epilepsy. The transcription factor nuclear factor (erythroid-derived 2)-like 2 (Nrf2) has emerged in recent years as an attractive therapeutic approach targeting to upregulate the antioxidative defenses in the cell, to ameliorate the oxidative stress-induced damage. Thus, it is important to understand the characteristics of Nrf2 activation during epileptogenesis and epilepsy. Here, we studied the temporal, regional, and cell-type specific expression of Nrf2 in the brain, in a rat model of temporal lobe epilepsy.

**Results:**

Early after status-epilepticus, Nrf2 is mainly activated in the hippocampus and maintained during the whole period of epileptogenesis. Only transient expression of Nrf2 was observed in the cortex. Nevertheless, the expression of several Nrf2 antioxidant target genes was increased within 24 h after status-epilepticus in both the cortex and the hippocampus. We demonstrated that after status-epilepticus in rats, Nrf2 is predominantly expressed in neurons in the CA1 and CA3 regions of the hippocampus, and only astrocytes in the CA1 increase their Nrf2 expression.

**Conclusions:**

In conclusion, our data identify previously unrecognized spatial and cell-type dependent activation of Nrf2 during epilepsy development, highlighting the need for a time-controlled, and cell-type specific activation of the Nrf2 pathway for mediating anti-oxidant response after brain insult, to modify the development of epilepsy.

**Supplementary Information:**

The online version contains supplementary material available at 10.1186/s13578-022-00951-y.

## Background

More than 65 million people worldwide suffer from epilepsy, which imposes a tremendous psychological, social, and economical burden on patients, caregivers, and society [1–3]. Currently, a substantial number of anti-seizure medications (ASMs) have been approved by FDA for the treatment of epileptic seizures, these treatments only target the disease’s symptoms and fail to effectively prevent seizure development or permanently stop the occurrence of seizures [4–6]. Additionally, 35% of all epileptic patients are resistant to ASMs and continued to experience recurring unprovoked spontaneous seizures [7]. Drug resistance is a particular problem in patients with temporal lobe epilepsy (TLE), where seizures originate from the hippocampus, the most epileptogenic region of the brain [8]. Many of these epilepsies are acquired conditions following a brain insult including traumatic brain injury (TBI), prolonged seizure *i.e.,* status epilepticus, or stroke [9, 10]. There is accumulating evidence suggesting that oxidative stress (OS) plays a critical role in acute neurological injuries such as prolonged seizures [11], stroke [12], and TBI [13], as well as in neurodegenerative diseases such as Parkinson’s [14] and Alzheimer’s disease [15, 16].

The nuclear factor erythroid 2-related factor 2 (Nrf2), described as the “master regulator” of the anti-oxidant response, has recently emerged as an important therapeutic target for various diseases, including neurologic disorders [17]. Under physiological conditions, Nrf2 is sequestered by its repressor, Kelch-like ECH associating protein 1 (Keap1) in the cytosol [18]. Under oxidative stress or other disturbance in cellular homeostasis, Nrf2 is translocated to the nucleus, promotes the expression of crucial genes responsible for detoxification, antioxidant and anti-inflammatory genes, and orchestrates the protective response [19]. Initially, *in-vivo* studies showed that Nrf2 knockout mice exhibit high-frequency seizure with prolonged duration, increased hippocampal neuronal death, and mortality following kainate toxicity [20]. In 2013, Wang et al., observed elevated expression of Nrf2 and regulated genes in the hippocampus of kindling seizure rats. These observations highlight that oxidative stress is a key player behind kindled seizure and suggested the possibility of activation of Nrf2-ARE signaling axis in the hippocampus following seizure [21]. Interestingly in 2013, Mazzuferi et al.found elevated expression of Nrf2 at the mRNA level in the temporal lobe epilepsy patient-derived hippocampal tissue samples [22]. Furthermore, they showed progressively elevated levels of Nrf2 following pilocarpine-induced status epilepticus in mice model [22]. Interestingly, they also observed a similar pattern of increased expression for three Nrf2-regulated genes [22]. Altogether these findings strongly suggest the protective role of Nrf2 in epilepsy. Even though, Nrf2 has been reported for its protective role in various pathologies associated with oxidative stress [18, 23]. In epilepsy animal models, we have recently demonstrated that pharmacological Nrf2 activation following status epilepticus (SE) in rats is neuroprotective and suppresses the development of seizures [24]. When Nrf2 activation was combined with pharmacological NOX inhibition, the development of epilepsy was prevented in 70% of animals following SE. This combination therapy also modified the severity of epilepsy when administered to chronic epileptic animals [25]. We also showed that when overactivation of Nrf2 following SE significantly decreased neuronal cell death in rats [26]. Several previous studies, conducted on neurological disorders, showed the restricted activation and expression of Nrf2 in astrocytes [27–33] while suppressed or weak expression in neurons [33–35]. In response to inducers, a robust Nrf2 activation is often observed in primary neuronal cultures [34, 36–38]. Neuronal cultures always contain at least a minor population of astrocytes [39]. When specific markers are used, it is clear that strong Nrf2 activation is restricted to the astrocytes in these cultures [36, 38, 40–42]. Although Nrf2 is more often expressed in astrocytes in cell cultures, neuronal Nrf2 activation is apparent in humans and mice [43]. The fact that Nrf2 inducers activate Nrf2 mainly in astrocytes and not in neurons in vitro suggests that their protective effects in animal models of neurodegeneration are due to Nrf2 activation specifically in astrocytes [27, 28]. Yet, the available evidence is mixed, and the spatio-temporal and cell type(s) specific dynamic expression of Nrf2 in the context of the chronic seizure model has not been studied. In this present study, we investigated the spatio-temporal and cell-type-specific expression pattern of Nrf2 following kainic acid-induced status epilepticus, a well-validated model of TLE [44]. While in the cortex we observed a slight increase in the Nrf2 mRNA levels only 24 h after SE, these were increased in the hippocampus for up to 2 weeks following SE. Furthermore, we observed a significant increase in the Nrf2-associated genes immediately after termination of SE that lasted for 24 h after. At the late epileptic phase, only the protein levels of Nrf2 and its prototypic gene were increased in the cortex and the hippocampus. Moreover, our immunohistochemical investigations showed that following SE, Nrf2 expression is predominantly increased in the neurons of CA1 region of the hippocampus, and little increase was detected in the CA3 and no change was observed in the cortex. In the astrocytes, the expression of Nrf2 was increased only in the CA1 region with no change in the CA3 and cortex. These results suggest that following a stimulus such as SE, Nrf2 pathway is activated in a region specific, time-dependent and cell-specific manner.

## Materials and methods

### Animals

Animal experiments were performed on male and female Sprague–Dawley rats (weight 165-230 g), Harlan strain of the Hebrew University of Jerusalem, Israel. Rats were housed under standard conditions (23 ± 1 °C; 50–60% humidity; 12-h light/dark cycle) in groups with free access to a chow diet and water. All procedures described herein were conducted according to the Association for Assessment and Accreditation of Laboratory Animal Care International and approved by the Institutional Animal Care and Use Committee of the Hebrew University of Jerusalem (Approval No.: MD-20–16254-5). All animals were subjected to acclimatization at the animal house for at least 3–7 days before experimental use.

### Induction of seizure

To induce seizures in SD rats, we used the previously described protocol of kainic acid injection [45]. Briefly, rats were administered intraperitoneally with the 5 mg/kg/hr dose of kainic acid (Hello Bio, Bristol, UK) and keep monitoring continuously for convulsive motor seizures (scored as per a modified Racine’s scale [46, 47] until repeated class III, IV, or V seizures were evoked. To avoid excessive toxicity and mortality, the subsequent dose of injections was reduced to 2.5 mg/kg if an animal began showing excessive inactivity or excessive activity like exaggerated running or jumping. The endpoint for kainic acid administration was either when the animals had a class V seizure (excessive rearing with concomitant forelimb clonus and falling) or when the cumulative dose of KA reached 45 mg/kg. Animals were sacrificed at specific time points: 0, 24, 48, and 72, hours as well as 1, 2, 6, and 12 weeks after status epilepticus.

### Real time-polymerase chain reaction

The expression of Nrf2 and its target genes [*i.e.,* NQO1 [NAD(P)H: quinone oxidoreductase 1]; HO-1 (Heme oxygenase 1); GCLC1 (Glutamate-Cysteine Ligase Catalytic Subunit); Srxn (Sulfiredoxin); and CAT-2 (Catalase)] at mRNA level were estimated using quantitative RT-PCR. Specific primers were designed for each target gene using Primer Express Software 3.0 (Applied biosystem) by Gen-Bank sequence (Table 1).

**Table 1:** Primers used in real-time RT-PCR

Name	Accession number	Forward	Reverse
Nrf2	NM_031789	GCAACTCCAGAAGGAACAGG	GGAATGGCTCTCTGCCAAAAGC
NQO1	NM_017000	GTTTGCCTGGCTTGCTTTCA	ACAGCCGTGGCAGAACTATC
HO-1	NM_012580	ACAGGGTGACAGAAGAGGCTAA	CTGTGAGGGACTCTGGTCTTTG
Srxn1	NM_001047858	AATAGTGAGGTCACCAGCTTC	CAGACAGTATGAGTCCTGGTTG
GCLC-1	NM_012815	GAGTAGAGTTCCGACCAAT	GCTCCTGTGCCACTTTCA
CAT-2	NM_012520	CCTGACATGGTCTGGGACTT	CAAGTTTTTGATGCCCTGGT
GAPDH	NM_017008	GACATGCCGCCTGGAGAAAC	AGCCCAGGATGCCCTTTAGT

TRI reagent (Sigma-Aldrich, St. Louis, MO, USA) was utilized to isolate total RNA from rat cortex and hippocampus tissue samples. Extracted RNAs were further assessed for their purity and concentration by using a Nanodrop spectrophotometer (Nanodrop Technologies, Thermo, Waltham, MA, USA) and the ratio of 260:280 was between 1.8 to 2.0. then complementary DNA (cDNA) was synthesized by using 1000 ng of RNA per sample (as a template), oligo-dT_15_ primers, and GoScript Reverse Transcription System (Promega). Expression of Nrf2 and its target genes were determined using SYBER Green (PerfeCTa SYBR Green FastMix, Quantabio) and RT-PCR (CFX version 3.1, Bio-Rad) instrument. The master mix reaction was prepared for 15 μl per sample that include (7.5 μl of SYBR Green, 3 μl of primer (500 nM each), 1.5 μl of RNase free water, and 3 μl of cDNA template) and was subjected to RT- PCR reaction cycles. Reaction cycles comprised of initial incubation at 95 °C for 10 min, followed by 40 cycles of denaturation for 5 s at 95 °C, 15 s at 60 °C, and a final step of 5 s at 65 °C and 30 s at 95 °C. All reactions were performed in duplicates. For each sample, gene expression of GAPDH was also determined as a control. The results were calculated and expressed concerning the control group, normalized to 1, and the Pfaffl et al. [48] mathematical method was used to calculate the relative expression of each gene.

### Western blotting

Animals were deeply anesthetized with an intraperitoneal administration of ketamine 100 mg/kg/xylazine 10 mg/kg, then sacrificed at the respective time points. Brains were collected immediately, and cortical and hippocampal tissue samples were dissected out and were further subjected to homogenization and lysis using RIPA buffer (Thermo Scientific) with freshly added protease inhibitor PI-(III) cocktail (Sigma). Protein concentrations were estimated using Pierce™ BCA Protein Assay Kit (Thermo). An equivalent amount of each sample *i.e.,* 25 μg was loaded onto each well of 10–12% SDS-PAGE gel, and resolved proteins were transferred to the nitrocellulose membrane (Bio-rad, Hercules, CA, USA). Protein transferred membranes were subjected to blocking either with 5% skim-milk for Nrf2 and NQO1 or 5% BSA for β actin followed by overnight at 4 °C incubation with either of primary antibodies against Nrf2 (1:1000, ab31163), NQO1 (1:1000, ab34173) and β actin (1:2000, ab8226). Primary antibody incubated membranes were washed three times (10 min each) with PBST (0.1% Tween-20 in PBS) and subjected to 2 h of dark incubation with HRP conjugated secondary antibody at room temperature. Incubated membranes were washed (as mentioned above) under dark conditions. Immunological complexes were visualized with electrochemiluminescence (ECL, Bio-Rad) and band intensities were analyzed with Image Lab Software (Bio-Rad, Hercules, CA, USA). The Nrf2 and NQO1 protein band intensities were normalized against β-actin band intensities values, and the average of the sham group was normalized to 1. The estimated values of all time points were expressed concerning the sham-operated group.

### Immunohistochemistry and microscopy

At 1 week following kainic acid-induced status epilepticus, rats were deeply anesthetized with an intraperitoneal injection of ketamine 100 mg/kg/xylazine 10 mg/kg, then cardially perfused with 4% PFA (Paraformaldehyde, Sigma) in phosphate buffer saline (PBS). Brain samples were recovered rapidly and stored overnight at 4 °C in 4% PFA-PBS solution then transferred to the 10–20–30% sucrose gradient for cryoprotection for the next 3–4 days until tissue sank. Cryoprotected brains were fixed in O.C.T. compound (Scigen) and stored at − 80 °C for further use. Each brain sample was cut with a cryostat (Leica CM1950) at − 20 °C and fixed on poly-L-lysine coated slides (Thermo Fisher Scientific) and 25 µm coronal sections were selected from the cortex and hippocampus subjected to air drying at room temperature for 2–3 h. Sections were circled with water repellent pen (Dako pen, Agilent) and permeabilized by 0.2% Triton X-100 (Sigma) in PBS for 30 min, then sections were subjected to blocking using 7% goat serum (Sigma) for 2–3 h, then were washed three times 10 min each with PBS. Washed sections were incubated at 4 °C overnight with rabbit primary antibody against Nrf2 (1:100, ab31163), and mouse primary antibody against NeuN (1:500, ab104224) or chicken primary antibody against GFAP (1:1000, ab4674) in the solution of PBS, 0.1% Triton X-100 and 1% BSA. Sections were further washed three times with PBS as mentioned above and were incubated at room temperature for 2 h in dark condition with Alexa Fluor 488 goat anti-mouse secondary antibody (1:500; ab150117, Abcam), Alexa Fluor-568 goat anti-rabbit secondary antibody (1:500; ab 175,695, Abcam) and Alexa Fluor-647 goat anti-chicken (1:500; ab150175, Abcam). Sections were further subjected for washing as mentioned above and mounted with Vectashield and DAPI (4', 6-diamidino-2-phenylindole) mounting medium (Vector Labs). Images were captured at a resolution of 1024 × 1024 with a Nikon confocal A1R microscope coupled with 20 × and 40 × objective lens. Images were analyzed using ImageJ software in a manual cell-counting image-based tool. Nrf2 + /NeuN + and Nrf2 + /GFAP + cells were counted on three ROI from each section, and 2–3 sections from each animal by investigators blinded to treatments. Cell densities were normalized with sham animals and expressed as a percentage of Nrf2 + /NeuN + ; Nrf2/GFAP + per area (mm^2^).

### Statistical analysis

The statistical analyses were performed using GraphPad Prism v9.3.1 software (GraphPad, USA). All data are expressed as the mean ± standard error of the mean (SEM). All data were analyzed by ordinary One-way analysis of variance (ANOVA) followed by Dunnett *post-hoc* test. Statistical difference between the two groups was assessed by Mann–Whitney U test. A p-value smaller or equal to 0.05 was considered statistically significant. Sample sizes were determined based on our previous experience with calculating experimental variability. The number of animals utilized is specified in the respective figure’s legend.

## Results

### Nrf2 has a delayed activation in the cortex following status epilepticus

**Fig. 1:**
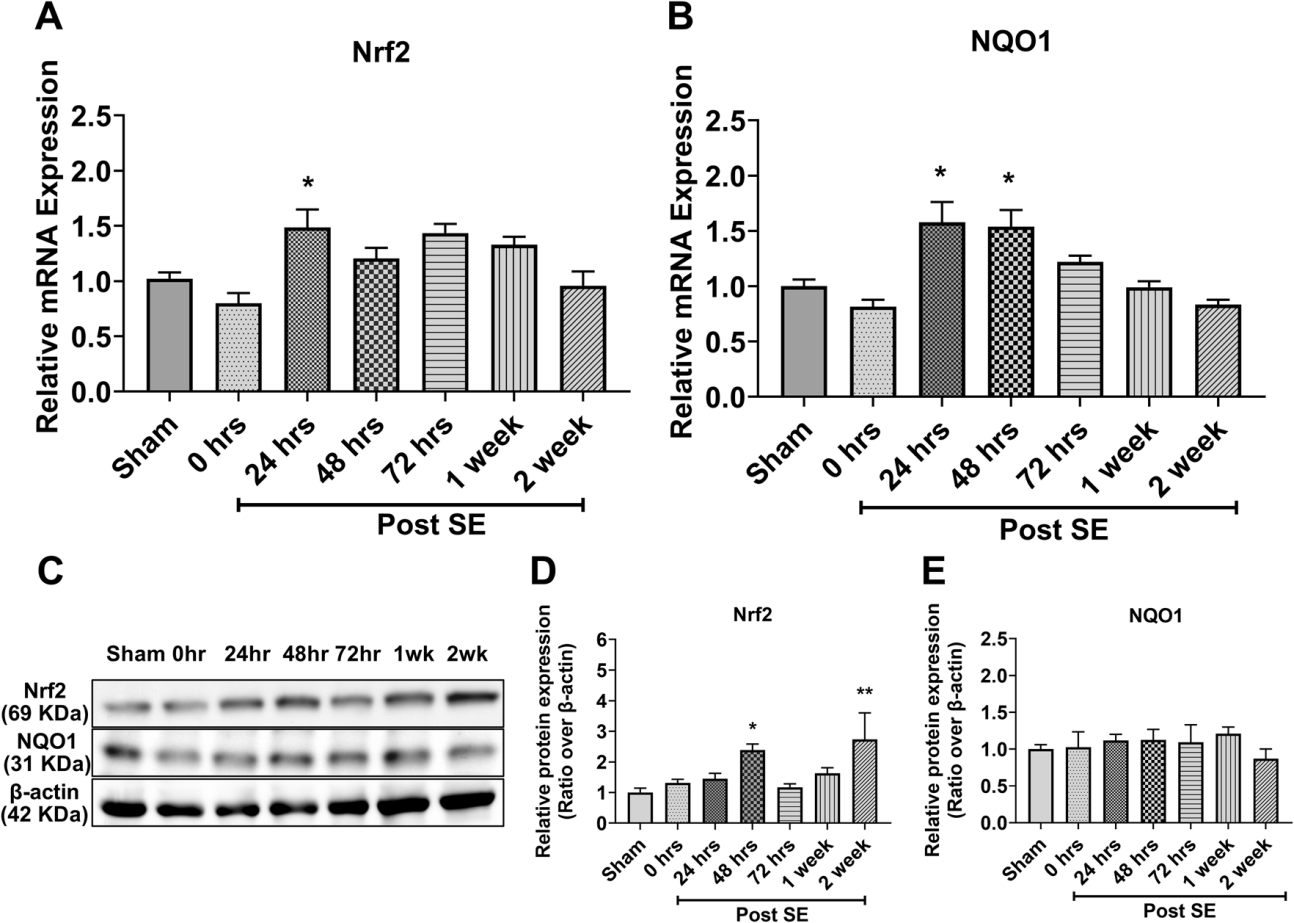
The temporal expression of Nrf2 and NQO1 in the cortex after SE. **A**, **B** Relative expression of mRNA levels of Nrf2 (**A**) and its downstream gene NAD(P)H: quinone oxidoreductase 1 (NQO1) (**B**) in the cortex at different time points after kainic acid-induced status epilepticus (SE) (n = 5 rats for each time point). **C** Nrf2 and NQO1 expression in the cortex following kainic acid induced-SE at the protein level using western blot analysis. **D**, **E** Relative protein quantification of each time point (n = 5) for Nrf2 (**D**) and NQO1 (**E**). Results are expressed as relative protein expression and reported as mean ± SEM. *P < *0.05 analyzed by one-way ANOVA followed by Dunnett* post-hoc *test*

We used the kainic acid-induced post-SE (KA-SE) model of TLE to demonstrate the spatial and temporal expression pattern of Nrf2 in the brain. The KA-SE model is a well-validated model of TLE and lends itself to investigating the epileptogenic process in pharmacoresistant epilepsy [44, 49]. We performed a time-course study to determine the Nrf2 as well as its target genes expression in the cortex and the hippocampus isolated from rats at 0, 24, 48, and 72 h, as well as 1-, and 2- week post-KA-SE (n = 5 rats/time point). In the cortex, a significant increase in the mRNA levels of Nrf2 was observed 24 h after SE (Fig. 1A). A slight increase was also observed during the first week after SE, however, this didn’t reach significance (Fig. 1A). However, we observed that expression of Nrf2 at protein level slightly increases at 48 h then return to basal level and at week 2 after SE Nrf2 shows threefold increase (Fig. 1C, D). Similarly, we measured the expression of NQO1, a prototypic target of Nrf2, and observed a significant increase at mRNA level at 24 and 48 h after SE (Fig. 1B), while at the protein level we didn’t find any significant changes up to 2-weeks after SE (Fig. 1C, E).

### Nrf2 shows prolonged activation in the hippocampus following status epilepticus

**Fig. 2:**
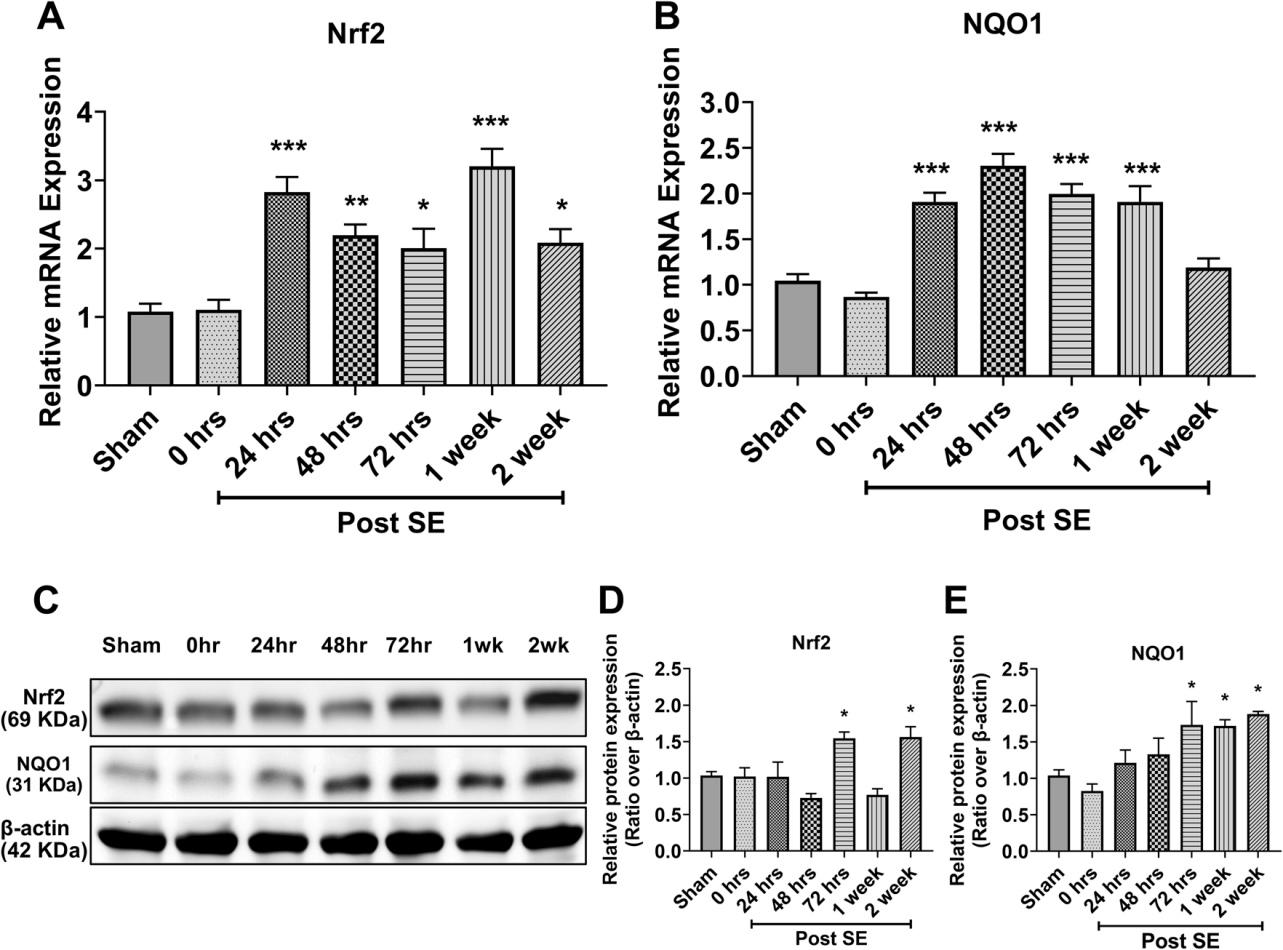
The temporal expression of Nrf2 and NQO1 in the hippocampus after SE. **A**, **B** Relative expression of mRNA levels of Nrf2 (A) and its downstream gene NAD(P)H: quinone oxidoreductase 1 (NQO1) (**B**) in the hippocampus at different time points after kainic acid-induced status epilepticus (SE) (n = 5 rats for each time point). **C** Nrf2 and NQO1 expression in the hippocampus following kainic acid induced-SE at the protein level using western blot analysis. **D**, **E** Relative protein quantification of each time point (n = 5) for Nrf2 (**D**) and NQO1 (**E**). Results are expressed as relative protein expression and reported as mean ± SEM. *P < 0.05, **P < 0.01, ***P < *0.001 analyzed by one-way ANOVA followed by Dunnett* post-hoc *test*

Next, we tested the possibility of region-dependent expression of Nrf2 following SE. We, therefore, determined the expression of Nrf2 and its prototypic target gene NQO1 in the hippocampus at different time points after SE. Unlike in the cortex, the Nrf2 mRNA levels in the hippocampus were significantly increased 24 h after SE and were maintained for up to 2 weeks (Fig. 2A). A similar pattern was observed also for NQO1 (Fig. 2B). Interestingly, we observe that expression of Nrf2 at protein level significantly increases at 72 h and 2 weeks after SE (Fig. 2C, D). However, the expression of NQO1 at protein level shows significantly increase at 72 h after SE and maintained up to 2 weeks (Fig. 2C–E).

### Nrf2-regulated genes display an early and transient expression in the cortex and the hippocampus following status epilepticus

**Fig. 3:**
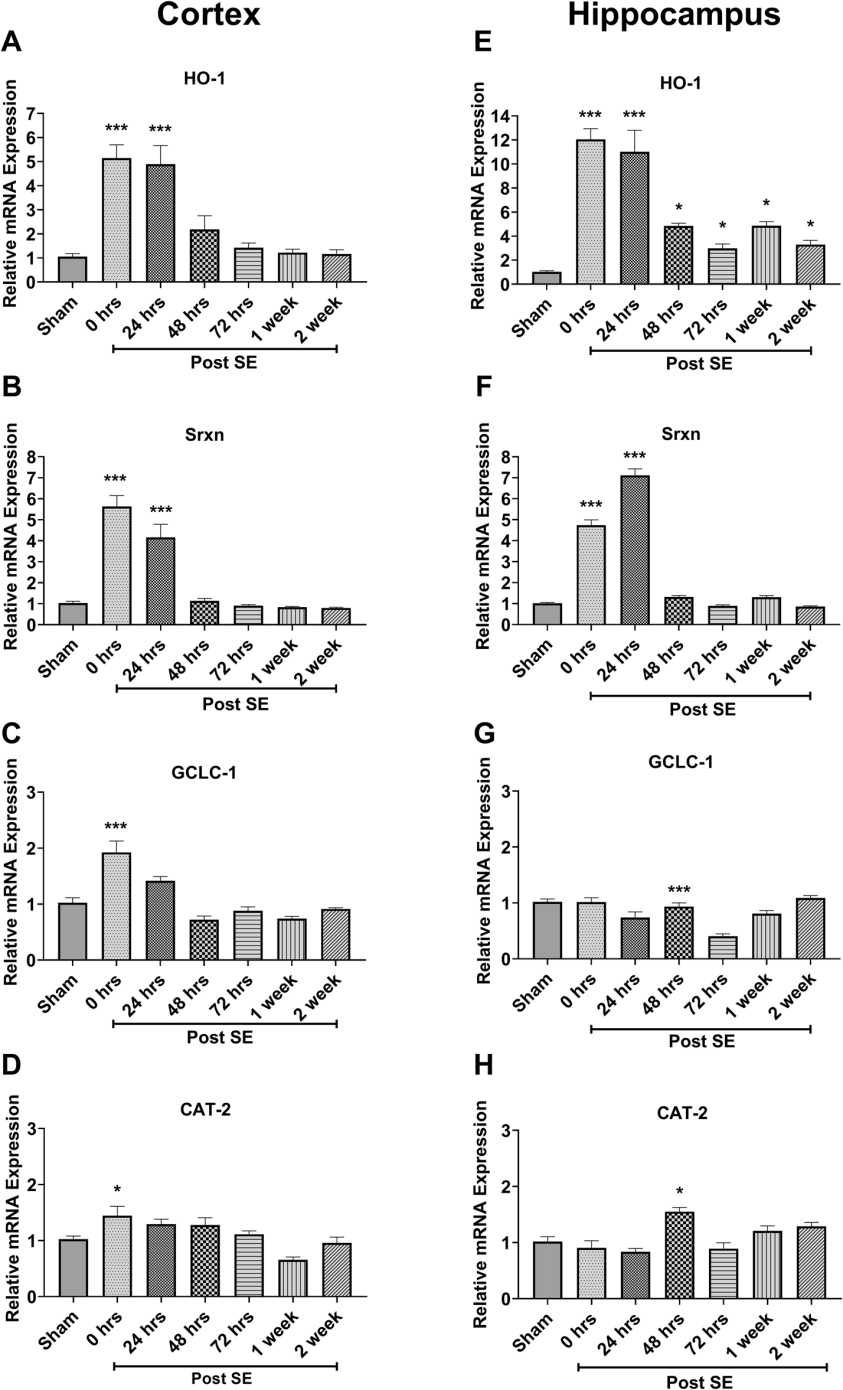
The expression of Nrf2 downstream target genes following SE. The temporal expression at mRNA level using RT-PCR of Nrf2 downstream target genes: (**A**) heme oxygenase-1 [HO-1], (**B**) Sulfiredoxin [Srxn], (**C**) Glutamate-Cysteine Ligase Catalytic Subunit 1 [GCLC1], and (**D**) Catalase [CAT-2] in the cortex (**A**–**D**) and the hippocampus (**E**–**H**) following kainic acid induced-SE. Results are expressed as relative mRNA expression and reported as mean ± SEM. *P < 0.05, **P < 0.01, ***P < *0.001 analyzed by one-way ANOVA followed by Dunnett* post-hoc* test*

Under stimulus by oxidative stress, Nrf2 translocates to the nucleus and activates the expression of many crucial genes responsible for detoxification and antioxidant activity [50]. Therefore, we asked whether kainic acid induced-SE will trigger any changes in the expression of four crucial target genes of Nrf2 in the cortex and hippocampus. We measured the expression of Heme oxygenase 1 (HO-1), Glutamate cysteine ligase catalytic (GCLC1), Sulfiredoxin 1 homolog (Srxn), and Catalase 2 (CAT-2) genes at several time points after SE. In the cortex, we found that the expression of all measured target genes was increased in comparison to sham animals at 0 h after SE (i.e. immediately after 2 h of SE; Fig. 3A–D). Interestingly, the levels of HO-1 and Srxn, but not GCLC-1 and CAT-1, were maintained for an additional 24 h after SE and then return to basal level (Fig. 3A–D). In the hippocampus, the mRNA levels of HO-1 were significantly increased, compared to the sham group, immediately after SE termination (i.e. 0 h), and this increase was maintained for up to 2 weeks after SE (Fig. 3 E). The mRNA levels of Srxn in the hippocampus displayed a similar pattern to that in the cortex, i.e. increase during the first 24 h after SE, then return to basal levels (Fig. 3F). Unlike in the cortex, the mRNA levels of GCLC1 did not show any significant changes within the 2 weeks, while a slight but significant increase in the levels of CAT-2 was observed at 48 h after SE (Fig. 3G, H).

Overall, a similar pattern of temporal expression at the protein level was detected in the cortex (Additional file 1: Fig. S2) and the hippocampus (Additional file 1: Fig. S3) for HO-1, Srxn, GCLC-1 and CAT-2.

### Nrf2 is increased in the cortex and the hippocampus at the chronic-epileptic stage

**Fig. 4:**
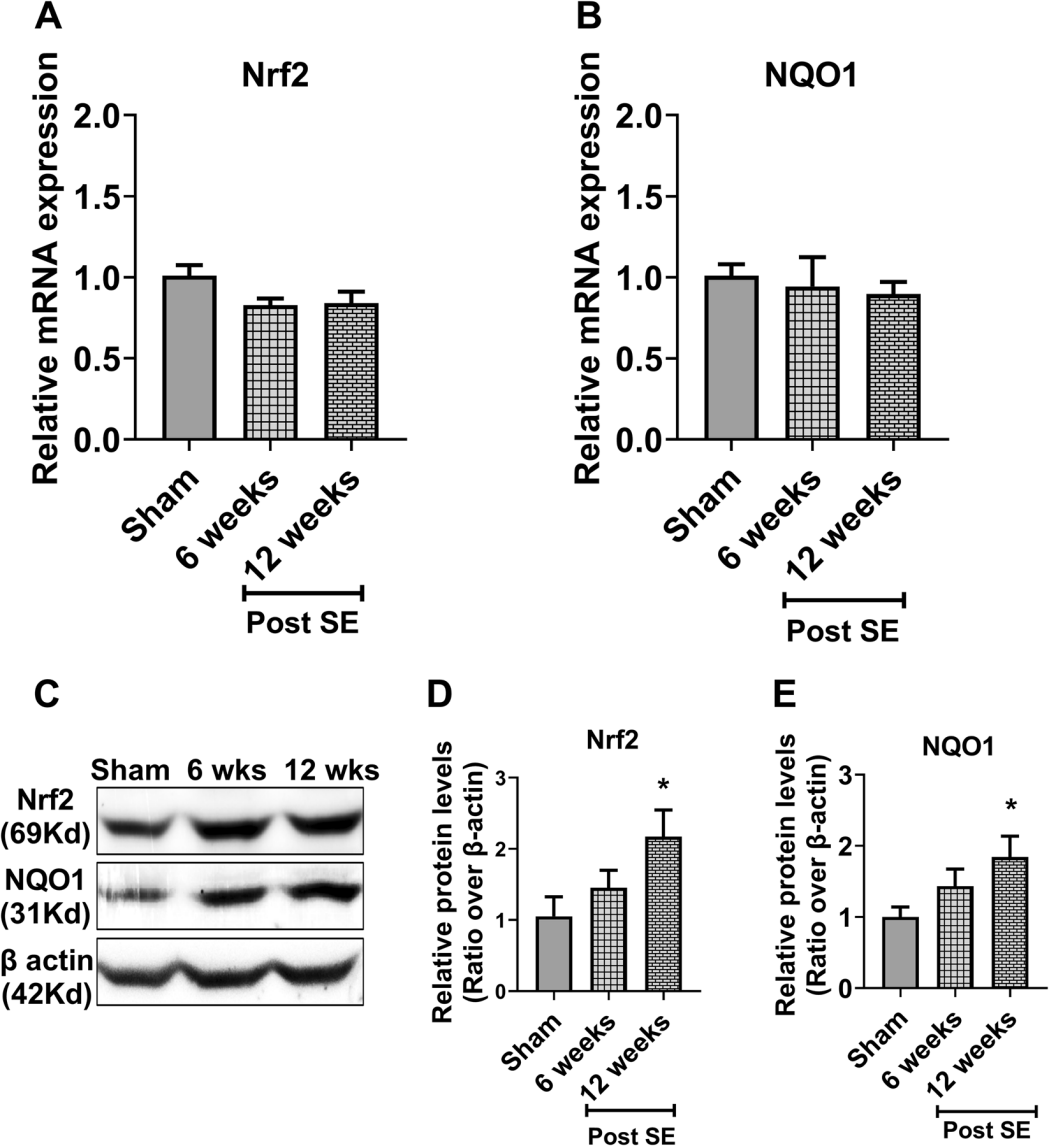
The expression of Nrf2 and NQO1 in the cortex during the late epileptic phase. **A**, **B** Relative expression of mRNA levels of Nrf2 (**A**) and its downstream gene NAD(P)H: quinone oxidoreductase 1 (NQO1) (B) in the cortex at 6- and 12-weeks after kainic acid-induced status epilepticus (SE) (n = 5 rats for each time point). **C** Nrf2 and NQO1 expression in the cortex at the protein level using western blot analysis. **D**, **E** Relative protein quantification of each time point (n = 5) for Nrf2 (D) and NQO1 (**E**). Results are expressed as relative protein expression and reported as mean ± SEM. *P < *0.05 analyzed by one-way ANOVA followed by Dunnett* post-hoc *test*

**Fig. 5:**
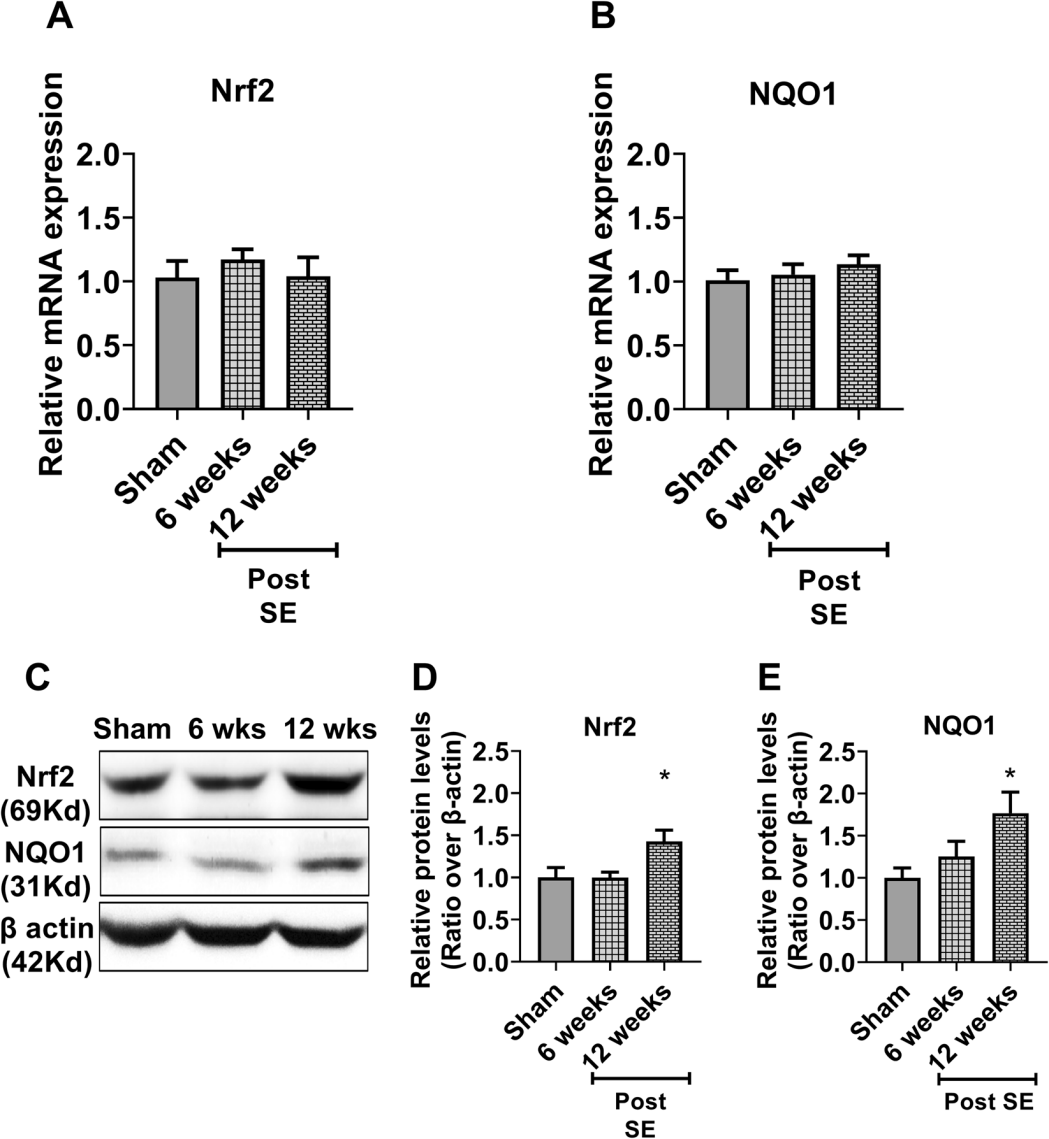
The expression of Nrf2 and NQO1 in the hippocampus during the late epileptic phase. **A**, **B** Relative expression of mRNA levels of Nrf2 (**A**) and its downstream gene NAD(P)H: quinone oxidoreductase 1 (NQO1) (**B**) in the hippocampus at 6- and 12-weeks after kainic acid-induced status epilepticus (SE) (n = 5 rats for each time point). **C** Nrf2 and NQO1 expression in the hippocampus at the protein level using western blot analysis. **D**, **E** Relative protein quantification of each time point (n = 5) for Nrf2 (**D**) and NQO1 (**E**). Results are expressed as relative protein expression and reported as mean ± SEM. *P < *0.05 analyzed by one-way ANOVA followed by Dunnett* post-hoc *test*

We further asked whether the expression of Nrf2 and its target genes, in the cortex and the hippocampus, display dynamic changes during the late epileptic stage. For this, we measured the mRNA and protein levels of Nrf2 and its prototypic gene NQO1, as well as the mRNA levels of other several target genes at 6- and 12 weeks following KA-SE, at a time rats usually became epileptic. While in the cortex we observed no changes in the mRNA levels of Nrf2 and NQO1 (Fig. 4 A–B), their protein expression was progressively increased compared to sham animals, with significantly higher levels at 12 weeks post SE (Fig. 4C–E). Importantly, a similar pattern of expression in both the mRNA and protein levels of Nrf2 and NQO1 was observed in the hippocampus (Fig. 5).

Interestingly, at these epileptic time points, we observed a trend of increase in the mRNA levels of HO-1 in both the cortex and the hippocampus, while we didn’t observe any significant changes in the levels of Srxn, GCLC-1 or CAT-2, neither in the cortex nor in the hippocampus (Additional file 1: Fig. S1).

### Expression of Nrf2 is predominant in hippocampi-neurons following SE

**Fig. 6:**
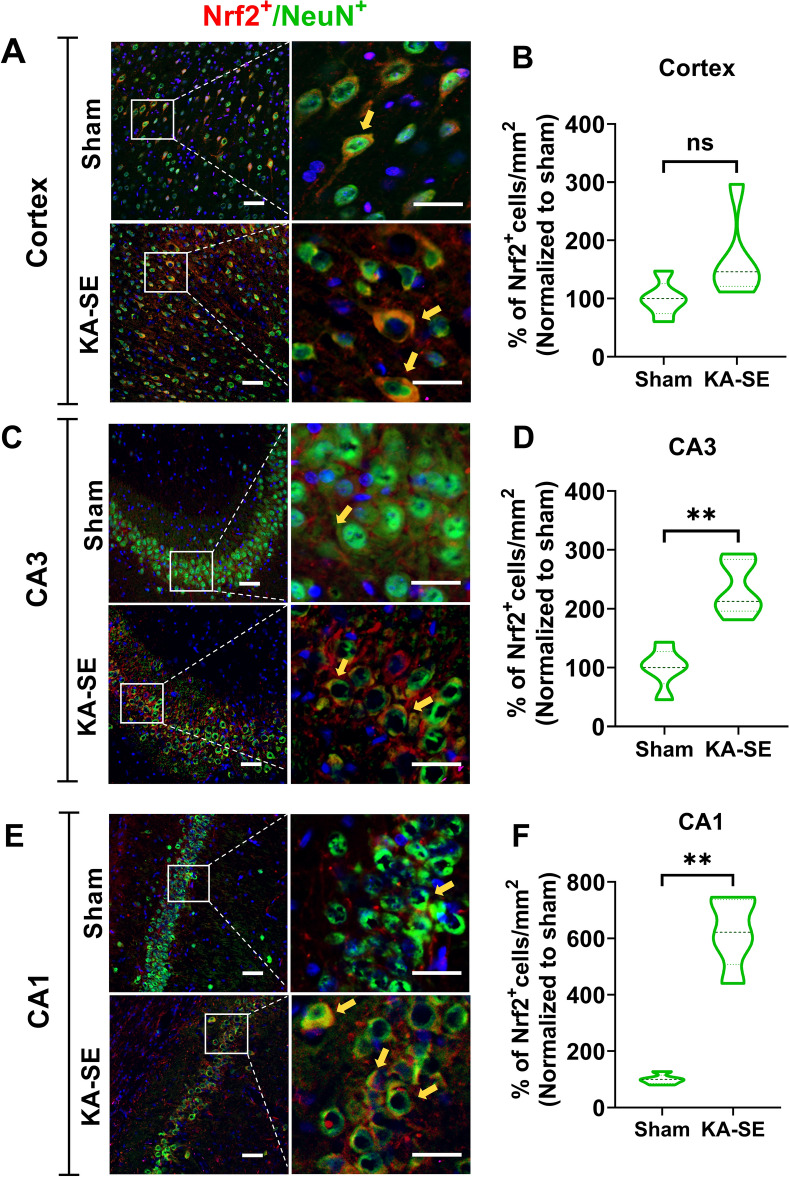
Nrf2 expression in neurons in the cortex and hippocampus at 1-week after SE. Representative fluorescent images of rat cortex (**A**), CA3 (**C**), and CA1 (**E**) regions of the hippocampus from sham animals and kainic acid induced-SE animals, illustrating colocalization of Nrf2 (red) with NeuN (green) and DAPI (blue). Scale bar = 50 µm (left panels) and 15 µm (right panels, zoomed view). **B**, **D**, **F**: The corresponding quantification of the number of cells double-positive for NeuN and Nrf2 in 1 mm^2^ of tissue, and normalized to the sham group. N = 5 rats/group. Results were expressed as mean ± SEM. **P < 0.01 compared to sham group by Mann–Whitney U test. B: p = 0.0556; D: p = 0.0079; F: p = 0.0079

**Fig. 7:**
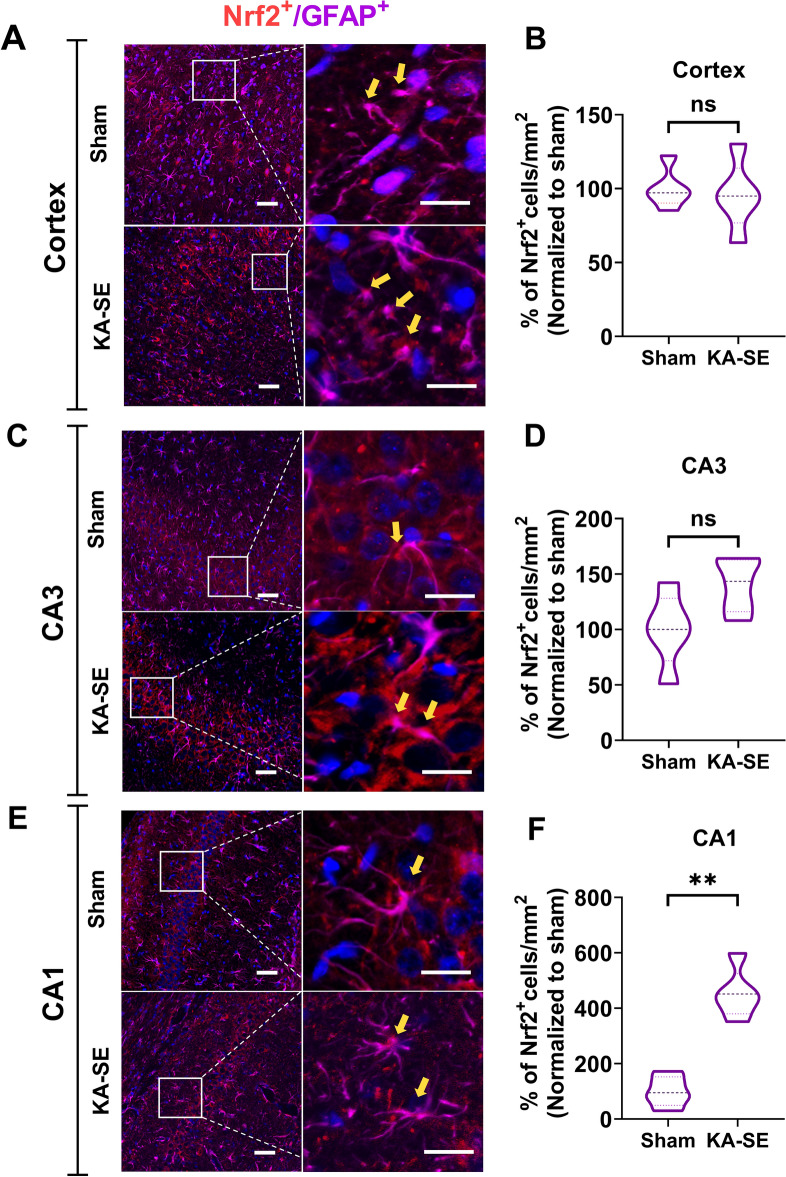
Nrf2 expression in astrocytes in the cortex and hippocampus at 1-week after SE. Representative fluorescent images of rat cortex (**A**), CA3 (**C**), and CA1 (**E**) regions in the hippocampus from sham animals and kainic acid induced-SE animals, illustrating colocalization of Nrf2 (red) with GFAP (purple) and DAPI (blue). Scale bar = 50 µm (left panels) and 15 µm (right panels, zoomed view). **B**, **D**, **F**: The corresponding quantification of the number of cells double-positive for GFAP and Nrf2 in 1 mm^2^ of tissue, and normalized to the sham group. N = 5 rats/group. Results were expressed as mean ± SEM. **P < 0.01 compared to sham group by Mann–Whitney U test. B: p = 0.6905; D: p = 0.0556; F: p = 0.0079

Several previous *in-vitro* and *in-vivo* studies on different disease models showed that activation of Nrf2-regulated gene expression was restricted predominantly to the astrocytes and only weak or basal expression was observed in the neurons [27–32, 34, 35]. Therefore, we ask if prolonged epileptic seizure (i.e., SE) can affect the expression of Nrf2 in neurons and astrocytes in a cell-type-dependent manner. We determined the Nrf2 expression in neuronal and astrocytic populations with the cell-type specific markers NeuN and GFAP, respectively, in both the cortex and the hippocampus of KA-SE rats using immunohistochemical staining, and we quantified the percentage of Nrf2 expressing neurons (Fig. 6) and astrocytes (Fig. 7). In parallel, we also demonstrate the spatial expression of Nrf2 in the cortex (Figs. 6A, B and 7A, B) as well as in the CA3 (Figs. 6 C, D and 7 C, D) and CA1 (Figs. 6E, F and 7E, F) area of the hippocampus. At 1-week after SE, the number of Nrf2 + /NeuN + cells was slightly increased by 167% in the cortex; however, this didn’t reach significance (Fig. 6A, B). A more profound and significant increase in the number of NeuN + cells expressing Nrf2 was observed in the CA3 (234% vs. Sham) and the CA1 (622% vs Sham) areas of the hippocampus (Fig. 6 C–F). Interestingly, in the astrocyte population, we observed a significant increase in the number of cells expressing Nrf2 only in the CA1 (452% vs Sham; Fig. 7E, F). We didn’t observe any change in the Nrf2 + /GFAP + cells neither in the cortex (Fig. 7A, B) or in CA3 (Fig. 7C, D).

Further, we asked whether this predominant expression of Nrf2 in neurons is altered during chronic phase ep epilepsy. To this end, we determined the Nrf2 expression in neurons and astrocytes at 12 week after SE. In neuronal population, the number of Nrf2 + /NeuN + cells was increased in the cortex as well as the CA3 and CA1 regions of the hippocampus (Additional file 1: Fig. S6A), in accordance with the changes observed 1-week after SE.

In the astrocytes, while a significant increase in the number of cells expressing Nrf2 was observed only in CA1 at 1-week after SE, a significant increase in the Nrf2 + /GFAP + cells was observed in both CA1 and CA3 12-weeks after SE (Additional file 1: Fig. S6 B). In the cortex however, no change was detected in the number of astrocytes expressing Nrf2, in correspondence with the results 1-week after SE.

## Discussion

Nrf2 has been identified in recent years as a key transcription factor emerging as a master regulator of the cellular antioxidant response [51]. Activation of Nrf2 is protective against oxidative stress in several pathologies, including neurodegenerative and neurological disorders [23]. Previous studies have demonstrated that Nrf2 has a particular pattern of activation in each neurodegenerative disease. For instance, Nrf2 is activated in astrocytes in Parkinson’s disease [52], while in Alzheimer’s disease it is activated in astrocytes, neurons, and possibly microglia [53]. In epilepsy animal models, Nrf2 has been shown to increase in kindled animals [21], and overexpressing Nrf2 showed a neuroprotective effect following status epilepticus (SE) in mice [22]. We have recently shown that activation of Nrf2 can suppress the development of epilepsy in a rat TLE model alone or in combination with an exogenous antioxidant [24, 54].

The anti-oxidative capacity and protective response of the Nrf2 pathway seem to be dependent on the pre-existent level of activation in the different regions and cell types in the brain. Previous reports showed that Nrf2 activation is neuroprotective only in cells/regions with a low physiological expression of Nrf2 [55]. In tissue or cells with a high baseline expression of Nrf2 and a profound antioxidant activity, the expression of Nrf2 may not be further increased endogenously by the given stimulus, such as SE.

Nevertheless, the evidence for spatial and cell-type-specific activation of Nrf2 in epilepsy is minimal and unclear. The results from this study reveal a robust expression of Nrf2 in the hippocampus, but not in the cortex, at early stages after SE, which increases again at chronic epilepsy. Interestingly, a predominant expression was detected in the neurons, and only a weak expression was observed in the astrocytes.

The spatial and cell-type specific expression of Nrf2 after SE suggests that Nrf2 has complex signaling in different brain regions and neural cells during epileptogenesis and chronic epilepsy, providing insight into possible therapeutics based on different neural cells and time windows.

We have previously reported that the Nrf2 pathway is endogenously activated after the KA-induced SE model of epilepsy in rats [24]. We have demonstrated that glutathione (GSH) levels, produced via transcriptional activation of the antioxidant response element (ARE), were significantly increased in the cortex and the hippocampus after SE. Interestingly, when animals were treated with an Nrf2 activator, omaveloxolone, the GSH levels increased in a dose-dependent manner, with significantly higher levels in the hippocampus compared to the cortex. These data suggest that the functional capacity of the Nrf2 pathway is higher than that of the endogenous response to the SE. Moreover, exogenous activation of Nrf2 is an effective means of increasing the antioxidant capacity in the cell. It seems paradoxical that Nrf2 was activated to higher levels in the hippocampus than that in the cortex. Another interesting observation from a previous study is that GSH synthesis via the Nrf2 pathway can be more easily induced in the hippocampus. This might suggest that the brain has a fine-tuned system that shows differences between brain regions. These findings also show that the antioxidant systems are not homogenously activated throughout the brain, suggesting a “region-specific antioxidant defense”.

It is well-documented that the hippocampus is the area of the brain that is most vulnerable to seizures [56]. After KA-SE, a detectable neuronal cell loss is mainly observed in the CA1 and CA3 regions of the hippocampus [24]. To fight against oxidative stress-induced neuronal cell death, neurons have to utilize extrinsic antioxidant support derived from surrounding astrocytes [34, 35, 57].

Previously, Mazzuferi et al. (2013) demonstrated that Nrf2 and its antioxidant target genes are transiently increased following pilocarpine-induced SE in mice. However, whether Nrf2 has a regional and/or cell-specific expression following this model was not tested and remains unclear.

Here, we demonstrated for the first time that the response of the Nrf2 pathway is predominant in the hippocampus and that it is activated to less extent in the cortex. Moreover, we showed for the first time that after SE, Nrf2 is activated mainly in the CA1 of the hippocampus, in both neurons and astrocytes.

Interestingly, in cells that already express high levels of Nrf2, further induction of it may interact with another promotor element, propagating cell death [58].

Thus, it is important to understand the time course and cell-type specific expression of Nrf2 at different stages of epilepsy.

Translation efficiency of a given mRNA can be influenced by many factors, one is post-translational modification of Nrf2 that could account for differences in mass and stability of the two forms. Even though KA-SE mediated oxidative insult significantly induces Nrf2 expression at mRNA level at the same time point in both cortex (Fig. 1A) and hippocampus (Fig. 2A) at 24 h but the duration for induced expression of Nrf2 at mRNA levels like in cortex showed only transient induction at mRNA level at 24 h only while hippocampus showed prolonged induction from 24 h to two weeks (Fig. 2A) following KA-SE, suggesting a region-dependent effect of SE on hippocampus then cortex. Previous studies also highlighted that KA-induced SE exhibit higher neurotoxicity in the hippocampus. However, Nrf2 expression at the protein level (Fig. 1C, D) gradually increased up to 48 h then return and maintain its basal level for the next 1 week, suggesting the possibility that KA-SE mediated oxidative insult not only induces transient expression at the mRNA level but also promotes the Nrf2 stability at the protein level either by supporting Nrf2 escape from Keap1 mediated degradation through direct effect (unknown mechanism) or by induction of mRNA level as an initial trigger. Interestingly, we observed that NQO1, a prototypic target of Nrf2, exhibits a similar expression pattern to Nrf2 following KA-SE as mRNA levels increased significantly at the same time point in both cortex (Fig. 1B), and hippocampus (Fig. 2B) at 24 h with different duration of induced expression that maintained up to 48 h then return to the basal level (Fig. 1B), supporting our hypothesis that KA mediated oxidative insult promotes the Nrf2 stability at protein level which leads to a gradual increase of Nrf2 up to 48 h. Even though we did not observe any significant increase at the protein level of NQO1 in the cortex (Fig. 1C, E) suggesting the possibility that significant induction of NQO1 at the protein level requires prolonged activation of Nrf2. Also in our previous report, we observed that following Nrf2 induction using sulforaphane in KA-SE rats, expression of NQO1 is much lower compared to Nrf2 [26] indicating the possibility that significant induction of NQO1 requires prolonged activation of Nrf2. Moreover, KA-SE induced prolonged elevated expression of Nrf2, at mRNA (Fig. 2A) and protein level (Fig. 2C, D), in the hippocampus significantly induced the expression of NQO1 at mRNA (Fig. 2B) as well as protein level (Fig. 2C, E) supporting our hypothesis that a significant expression of NQO1 at protein level requires prolonged activation of Nrf2.

An interesting observation from our results is the time-dependent activation of Nrf2 after SE.

We and others have found that in the KA-SE model, the time for the emergence of the first spontaneous seizure after SE, which is usually referred to as epileptogenesis, is ~ 2 weeks. We, therefore, measured the expression of Nrf2 at several time points during epileptogenesis, i.e., within 2 weeks after SE. In agreement with previous results reported by Mazzuferi et al. [22], we observed that Nrf2 activation in the hippocampus increased during epileptogenesis, i.e. 2 weeks after SE, and peaked at 24–72 h after SE. In addition, given that Nrf2 can activate the expression of various antioxidant genes including NQO1, HO-1, Srxn1, and others, that helps to potentiate antioxidant response and provide resistance to oxidative insults [59]. Interestingly, in our present studies, we observed a pattern of expression for NQO1 in similitude to that of Nrf2 at 24 h in the hippocampus. Similarly, at this time point, the levels of HO-1 and Srxn1 were increasingly maintained, suggestive of antioxidant defense response to cells during epilepsy development.

Interestingly, previous studies reported that there is a sex difference in susceptibility to epileptic seizures in rats, taking into account that testosterone may increase and decrease seizure susceptibility in females and males, respectively [60, 61]. We, therefore, asked whether the Nrf2 pathway shows a gender associated expression after SE. Our results revealed that in the cortex, the expression of Nrf2 and its target genes NQO1 and HO-1 was higher in the females compared to male rats at the chronic phase of epilepsy (i.e. 6–12 weeks post-SE), but not during epileptogenesis (Additional file 1: Fig. S4). Interestingly, a similar behavior was detected in the hippocampus with increased expression of Nrf2, NQO1, HO-1 GCLC-1, and CAT2 in females than in males (Additional file 1: Fig. S5). These results suggest that Nrf2 activation may be affected by steroid hormones, including fluctuations in neurosteroids.”

In previous studies, it was found that neurons are more vulnerable to oxidative insults than astrocytes since they have weaker antioxidant defenses [62–64]. It was demonstrated that following treatments with Nrf2 activators in vitro, astrocytes respond more strongly to these treatments than neurons, suggesting that Nrf2 activation is restricted mainly to astrocytes.

An important role of astrocytes is to maintain physiological homeostasis within the CNS, partly by supporting the antioxidant system of neurons [65–67]. Previous studies found that Nrf2 activation is not present in pure primary neuronal cultures without astrocytes, supporting the hypothesis that activation of Nrf2 in neurons is highly dependent on the abundance of surrounding astrocytes [64].

In our present study, we found that Nrf2 expression was increased predominantly in neurons of the CA1 and CA3 regions of the hippocampus, and no significant increase was detected in the cortex. The expression of Nrf2 in the astrocytes was increased only in the CA1 region. Interestingly, in both neurons and astrocytes, there was a profound increase in the expression of Nrf2 in the CA1 region. Further studies are required to address the question of whether cells in the CA1 region have a lower basal expression of Nrf2 than other areas in the brain.

## Conclusion

In conclusion, despite the crucial neuroprotective role of Nrf2, a widespread and non-specific activation may affect key developmental processes and pathways. Therefore, as activation of the Nfr2 pathway in epilepsy holds great promise, our results in the present study highlight that a targeted, time-controlled, and cell-type specific activation of the Nrf2 pathway should be considered, for mediating anti-oxidant response and fine-tuning the balance between epilepsy-induced oxidative stress and Nrf2 activation.

## Supplementary Information

**Additional file 1: Figure S1.** Temporal mRNA expression of Nrf2 downstream target genes during late epileptic phase: (A) heme oxygenase-1 [HO-1], (B) Sulfiredoxin [Srxn], (C) Glutamate-Cysteine Ligase Catalytic Subunit 1 [GCLC1], and (D) Catalase [CAT-2] in the cortex (A-D) and the hippocampus (E-H) following kainic acid induced-SE (n=6 rats for each time point). Results are expressed as relative mRNA expression and reported as mean ± SEM. **P<0.01 analyzed by one-way ANOVA followed by Tukey’s post-hoc test. **Figure S2.** The temporal expression of Nrf2 target genes at protein level in the cortex after SE. A) The expression of Nrf2 target genes, HO-1, Srxn, GCLC-1, and CAT-2 in the cortex following kainic acid induced-SE at the protein level using western blot analysis. B-E) Relative protein quantification of each time point (n=6) for HO-1 (B), Srxn (C), GCLC-1 (D), and CAT-2 (E). Results are expressed as relative protein expression and reported as mean ± SEM. *P<0.05 analyzed by one-way ANOVA followed by Dunnett post-hoc *test.* **Figure S3.** The temporal expression of Nrf2 target genes at protein level in the hippocampus after SE. A) The expression of Nrf2 target genes, HO-1, Srxn, GCLC-1, and CAT-2 in the cortex following kainic acid induced-SE at the protein level using western blot analysis. B-E) Relative protein quantification of each time point (n=6) for HO-1 (B), Srxn (C), GCLC-1 (D), and CAT-2 (E). Results are expressed as relative protein expression and reported as mean ± SEM. *P<0.05 analyzed by one-way ANOVA followed by Dunnett post-hoc test. **Figure S4.** Male and female associated expression of Nrf2 and target genes in the cortex after SE. Comparison of male and female expression of mRNA levels of Nrf2 (A), and its target genes NQO1 (B), HO-1 (C), Srxn (D), GCLC-1 (E), and CAT-2 (F) in the cortex at different time points after kainic acid-induced status epilepticus (SE) (Male (n=3)/Female (n=3) rats for each time point). Results are expressed as relative expression of mRNA levels and reported as mean ± SEM. *P<0.05 analyzed by unpaired Student’s t-test. **Figure S5.** Male and female associated expression of Nrf2 and target genes in the hippocampus after SE. Comparison of male and female expression of mRNA levels of Nrf2 (A), and its target genes NQO1 (B), HO-1 (C), Srxn (D), GCLC-1 (E), and CAT-2 (F) in the hippocampus at different time points after kainic acid-induced status epilepticus (SE) (Male (n=3)/Female (n=3) rats for each time point). Results are expressed as relative expression of mRNA levels and reported as mean ± SEM. *P<0.05 analyzed by unpaired Student’s t-test. **Figure S6.** Nrf2 expression in neurons and astrocytes in the cortex and hippocampus at 12-weeks after SE. (A) Representative fluorescent images of rat cortex, CA3, and CA1 regions of the hippocampus (left panels) from animals after 12 weeks of kainic acid induced-SE, illustrating Nrf2 (red), NeuN (green), and DAPI (blue). Scale bar = 50 µm and 15 µm (zoomed view). The right panels represent bar charts summarizing the corresponding quantification of the number of cells double-positive for NeuN and Nrf2 in 1 mm^2^ of tissue, and normalized to the sham group. N=5 rats/group. Results were expressed as mean ± SEM. Cortex: p=0.0317, CA3: p=0.0079, CA1: p=0.0079 compared to sham group by Mann–Whitney U test. (B) Representative fluorescent images of rat cortex, CA3, and CA1 regions of the hippocampus (left panels) from animals after 12 weeks of kainic acid induced-SE, illustrating Nrf2 (red), GFAP (magenta), and DAPI (blue). Scale bar = 50 µm and 15 µm (zoomed view). The right panels represent bar charts summarizing the corresponding quantification of the number of cells double-positive for NeuN and Nrf2 in 1 mm^2^ of tissue, and normalized to the sham group. N=5 rats/group.

## Data Availability

The datasets used and/or analysed during the current study are available from the corresponding author on reasonable request.

## Abbreviations

Cat-2: Catalase 2
GAPDH: Glyceraldehyde-3-phosphate dehydrogenase
GCLC-1: Glutamate cysteine ligase catalytic
HO-1: Heme oxygenase-1
NQO1: NAD(P)H: quinone oxidoreductase 1
Nrf2: Nuclear factor erythroid 2-like 2
OS: Oxidative Stress
PTZ: Pentylenetetrazol
ROS: Reactive oxygen species
SE: Status Epilepticus
Srxn: Sulfiredoxin 1

## References

[1] Thijs RD, Surges R, O’Brien TJ, Sander JW (2019). Epilepsy in adults. Lancet.

[2] Falco-Walter J (2020). Epilepsy-definition, classification, pathophysiology, and epidemiology. Semin Neurol.

[3] Beghi E (2020). The epidemiology of epilepsy. Neuroepidemiology.

[4] Glauser T, Ben-Menachem E, Bourgeois B, Cnaan A, Chadwick D, Guerreiro C (2006). ILAE treatment guidelines: evidence-based analysis of antiepileptic drug efficacy and effectiveness as initial monotherapy for epileptic seizures and syndromes. Epilepsia.

[5] Glauser T, Ben-Menachem E, Bourgeois B, Cnaan A, Guerreiro C, Kalviainen R (2013). Updated ILAE evidence review of antiepileptic drug efficacy and effectiveness as initial monotherapy for epileptic seizures and syndromes. Epilepsia.

[6] French JA (2007). Refractory epilepsy: clinical overview. Epilepsia.

[7] Duncan JS, Sander JW, Sisodiya SM, Walker MC (2006). Adult epilepsy. Lancet.

[8] Avoli M (2007). The epileptic hippocampus revisited: back to the future. Epilepsy Curr.

[9] Pitkanen A, Immonen R (2014). Epilepsy related to traumatic brain injury. Neurotherapeutics.

[10] Banerjee PN, Filippi D, Allen HW (2009). The descriptive epidemiology of epilepsy-a review. Epilepsy Res.

[11] Pearson-Smith JN, Patel M (2017). Metabolic dysfunction and oxidative stress in epilepsy. Int J Mol Sci.

[12] Zhang R, Xu M, Wang Y, Xie F, Zhang G, Qin X (2017). Nrf2-a Promising therapeutic target for defensing against oxidative stress in stroke. Mol Neurobiol.

[13] Bhatti J, Nascimento B, Akhtar U, Rhind SG, Tien H, Nathens A (2017). Systematic review of human and animal studies examining the efficacy and safety of N-Acetylcysteine (NAC) and N-Acetylcysteine amide (NACA) in traumatic brain injury: impact on neurofunctional outcome and biomarkers of oxidative stress and inflammation. Front Neurol.

[14] Puspita L, Chung SY, Shim JW (2017). Oxidative stress and cellular pathologies in Parkinson’s disease. Mol Brain.

[15] Cheignon C, Tomas M, Bonnefont-Rousselot D, Faller P, Hureau C, Collin F (2018). Oxidative stress and the amyloid beta peptide in Alzheimer’s disease. Redox Biol.

[16] Wang X, Wang W, Li L, Perry G, Lee HG, Zhu X (2014). Oxidative stress and mitochondrial dysfunction in Alzheimer’s disease. Biochim Biophys Acta.

[17] Ma Q (2013). Role of nrf2 in oxidative stress and toxicity. Annu Rev Pharmacol Toxicol.

[18] Liu T, Lv YF, Zhao JL, You QD, Jiang ZY (2021). Regulation of Nrf2 by phosphorylation: consequences for biological function and therapeutic implications. Free Radic Biol Med.

[19] Baird L, Dinkova-Kostova AT (2011). The cytoprotective role of the Keap1-Nrf2 pathway. Arch Toxicol.

[20] Kraft AD, Lee JM, Johnson DA, Kan YW, Johnson JA (2006). Neuronal sensitivity to kainic acid is dependent on the Nrf2-mediated actions of the antioxidant response element. J Neurochem.

[21] Wang W, Wang WP, Zhang GL, Wu YF, Xie T, Kan MC (2013). Activation of Nrf2-ARE signal pathway in hippocampus of amygdala kindling rats. Neurosci Lett.

[22] Mazzuferi M, Kumar G, van Eyll J, Danis B, Foerch P, Kaminski RM (2013). Nrf2 defense pathway: experimental evidence for its protective role in epilepsy. Ann Neurol.

[23] Esteras N, Dinkova-Kostova AT, Abramov AY (2016). Nrf2 activation in the treatment of neurodegenerative diseases: a focus on its role in mitochondrial bioenergetics and function. Biol Chem.

[24] Shekh-Ahmad T, Eckel R, Dayalan Naidu S, Higgins M, Yamamoto M, Dinkova-Kostova AT (2018). KEAP1 inhibition is neuroprotective and suppresses the development of epilepsy. Brain.

[25] Shekh-Ahmad T, Lieb A, Kovac S, Gola L, Christian Wigley W, Abramov AY (2019). Combination antioxidant therapy prevents epileptogenesis and modifies chronic epilepsy. Redox Biol.

[26] Sandouka S, Shekh-Ahmad T (2021). Induction of the Nrf2 Pathway by sulforaphane is neuroprotective in a rat temporal lobe epilepsy model. Antioxidants.

[27] Masaki Y, Izumi Y, Matsumura A, Akaike A, Kume T (2017). Protective effect of Nrf2-ARE activator isolated from green perilla leaves on dopaminergic neuronal loss in a Parkinson’s disease model. Eur J Pharmacol.

[28] Jazwa A, Rojo AI, Innamorato NG, Hesse M, Fernandez-Ruiz J, Cuadrado A (2011). Pharmacological targeting of the transcription factor Nrf2 at the basal ganglia provides disease modifying therapy for experimental parkinsonism. Antioxid Redox Signal.

[29] Duan W, Zhang R, Guo Y, Jiang Y, Huang Y, Jiang H (2009). Nrf2 activity is lost in the spinal cord and its astrocytes of aged mice. Vitro Cell Dev Biol Ani.

[30] Vargas MR, Johnson DA, Sirkis DW, Messing A, Johnson JA (2008). Nrf2 activation in astrocytes protects against neurodegeneration in mouse models of familial amyotrophic lateral sclerosis. J Neurosci.

[31] Calkins MJ, Vargas MR, Johnson DA, Johnson JA (2010). Astrocyte-specific overexpression of Nrf2 protects striatal neurons from mitochondrial complex II inhibition. Toxicol Sci.

[32] Draheim T, Liessem A, Scheld M, Wilms F, Weissflog M, Denecke B (2016). Activation of the astrocytic Nrf2/ARE system ameliorates the formation of demyelinating lesions in a multiple sclerosis animal model. Glia.

[33] Liddell JR (2017). Are Astrocytes the predominant cell type for activation of Nrf2 in aging and neurodegeneration?. Antioxidants.

[34] Shih AY, Johnson DA, Wong G, Kraft AD, Jiang L, Erb H (2003). Coordinate regulation of glutathione biosynthesis and release by Nrf2-expressing glia potently protects neurons from oxidative stress. J Neurosci.

[35] Dringen R, Hirrlinger J. Glutathione pathways in the brain. 2003. 10.1515/BC.2003.059 12751781

[36] Calkins MJ, Jakel RJ, Johnson DA, Chan K, Kan YW, Johnson JA (2005). Protection from mitochondrial complex II inhibition in vitro and in vivo by Nrf2-mediated transcription. Proc Natl Acad Sci.

[37] Lee JM, Shih AY, Murphy TH, Johnson JA (2003). NF-E2-related factor-2 mediates neuroprotection against mitochondrial complex I inhibitors and increased concentrations of intracellular calcium in primary cortical neurons. J Biol Chem.

[38] Murphy TH, Yu J, Ng R, Johnson DA, Shen H, Honey CR (2001). Preferential expression of antioxidant response element mediated gene expression in astrocytes. J Neurochem.

[39] Noble M, Fok-Seang J, Cohen J (1984). Glia are a unique substrate for the in vitro growth of central nervous system neurons. J Neurosci.

[40] Calkins MJ, Townsend JA, Johnson DA, Johnson JA (2010). Cystamine protects from 3-nitropropionic acid lesioning via induction of nf-e2 related factor 2 mediated transcription. Exp Neurol.

[41] Kraft AD, Johnson DA, Johnson JA (2004). Nuclear factor E2-related factor 2-dependent antioxidant response element activation by tert-butylhydroquinone and sulforaphane occurring preferentially in astrocytes conditions neurons against oxidative insult. J Neurosci.

[42] Liddell JR, Lehtonen S, Duncan C, Keksa-Goldsteine V, Levonen AL, Goldsteins G (2016). Pyrrolidine dithiocarbamate activates the Nrf2 pathway in astrocytes. J Neuroinflam.

[43] Deighton RF, Markus NM, Al-Mubarak B, Bell KF, Papadia S, Meakin PJ (2014). Nrf2 target genes can be controlled by neuronal activity in the absence of Nrf2 and astrocytes. Proc Natl Acad Sci.

[44] Hellier JL, Patrylo PR, Buckmaster PS, Dudek FE (1998). Recurrent spontaneous motor seizures after repeated low-dose systemic treatment with kainate: assessment of a rat model of temporal lobe epilepsy. Epilepsy Res.

[45] Saadi A, Sandouka S, Grad E, Singh PK, Shekh-Ahmad T (2022). Spatial, temporal, and cell-type-specific expression of NADPH Oxidase isoforms following seizure models in rats. Free Radical Biol Med.

[46] Racine RJ (1972). Modification of seizure activity by electrical stimulation II Motor seizure. Electroencephalogr Clin Neurophysiol.

[47] Ben-Ari Y (1985). Limbic seizure and brain damage produced by kainic acid: mechanisms and relevance to human temporal lobe epilepsy. Neuroscience.

[48] Pfaffl MW (2001). A new mathematical model for relative quantification in real-time RT-PCR. Nucleic Acids Res.

[49] Bertoglio D, Amhaoul H, Van Eetveldt A, Houbrechts R, Van De Vijver S, Ali I (2017). Kainic acid-induced post-status epilepticus models of temporal lobe epilepsy with diverging seizure phenotype and neuropathology. Front Neurol.

[50] Kaspar JW, Niture SK, Jaiswal AK (2009). Nrf 2: INrf2 (Keap1) signaling in oxidative stress. Free Radical Biol Med.

[51] Heurtaux T, Bouvier DS, Benani A, Helgueta Romero S, Frauenknecht KBM, Mittelbronn M (2022). Normal and pathological NRF2 signalling in the central nervous system. Antioxidants.

[52] van Muiswinkel FL, de Vos RA, Bol JG, Andringa G, Jansen Steur EN, Ross D (2004). Expression of NAD(P)H:quinone oxidoreductase in the normal and Parkinsonian substantia nigra. Neurobiol Aging.

[53] Ramsey CP, Glass CA, Montgomery MB, Lindl KA, Ritson GP, Chia LA (2007). Expression of Nrf2 in neurodegenerative diseases. J Neuropathol Exp Neurol.

[54] Pauletti A, Terrone G, Shekh-Ahmad T, Salamone A, Ravizza T, Rizzi M (2019). Targeting oxidative stress improves disease outcomes in a rat model of acquired epilepsy. Brain.

[55] Linker RA, Lee DH, Ryan S, van Dam AM, Conrad R, Bista P (2011). Fumaric acid esters exert neuroprotective effects in neuroinflammation via activation of the Nrf2 antioxidant pathway. Brain.

[56] Vasilev DS, Tumanova NL, Kim KK, Lavrentyeva VV, Lukomskaya NY, Zhuravin IA (2018). Transient morphological alterations in the hippocampus after pentylenetetrazole-induced seizures in rats. Neurochem Res.

[57] Dringen R, Pfeiffer B, Hamprecht B (1999). Synthesis of the antioxidant glutathione in neurons: supply by astrocytes of CysGly as precursor for neuronal glutathione. J Neurosci.

[58] Zucker SN, Fink EE, Bagati A, Mannava S, Bianchi-Smiraglia A, Bogner PN (2014). Nrf2 amplifies oxidative stress via induction of Klf9. Mol Cell.

[59] Lim JH, Kim KM, Kim SW, Hwang O, Choi HJ (2008). Bromocriptine activates NQO1 via Nrf2-PI3K/Akt signaling: novel cytoprotective mechanism against oxidative damage. Pharmacol Res.

[60] Reddy DS, Thompson W, Calderara G (2021). Molecular mechanisms of sex differences in epilepsy and seizure susceptibility in chemical, genetic and acquired epileptogenesis. Neurosci Lett.

[61] Tan M, Tan U (2001). Sex difference in susceptibility to epileptic seizures in rats: importance of estrous cycle. Int J Neurosci.

[62] Fernandez-Fernandez S, Almeida A, Bolanos JP (2012). Antioxidant and bioenergetic coupling between neurons and astrocytes. Biochem J.

[63] Dringen R, Pawlowski PG, Hirrlinger J (2005). Peroxide detoxification by brain cells. J Neurosci Res.

[64] Bell KF, Al-Mubarak B, Martel MA, McKay S, Wheelan N, Hasel P (2015). Neuronal development is promoted by weakened intrinsic antioxidant defences due to epigenetic repression of Nrf2. Nat Commun.

[65] Karve IP, Taylor JM, Crack PJ (2016). The contribution of astrocytes and microglia to traumatic brain injury. Br J Pharmacol.

[66] Abbott NJ, Ronnback L, Hansson E (2006). Astrocyte-endothelial interactions at the blood-brain barrier. Nat Rev Neurosci.

[67] Abbott NJ (2002). Astrocyte-endothelial interactions and blood-brain barrier permeability. J Anat.

