# Supplementary material for: Nrf2 is predominantly expressed in hippocampal neurons in a rat model of temporal lobe epilepsy
Source: Cell Biosci. 2023 Jan 4;13:3. doi: 10.1186/s13578-022-00951-y (PMC9814517; doi:10.1186/s13578-022-00951-y)
Supplement: Supplementary file 1 — Additional file 1: Figure S1. Temporal mRNA expression of Nrf2 downstream target genes during late epileptic phase: (A) heme oxygenase-1 [HO-1], (B) Sulfiredoxin [Srxn], (C) Glutamate-Cysteine Ligase Catalytic Subunit 1 [GCLC1], and (D) Catalase [CAT-2] in the cortex (A-D) and the hippocampus (E-H) following kainic acid induced-SE (n=6 rats for each time point). Results are expressed as relative mRNA expression and reported as mean ± SEM. **P<0.01 analyzed by one-way ANOVA followed by Tukey’s post-hoc test. Figure S2. The temporal expression of Nrf2 target genes at protein level in the cortex after SE. A) The expression of Nrf2 target genes, HO-1, Srxn, GCLC-1, and CAT-2 in the cortex following kainic acid induced-SE at the protein level using western blot analysis. B-E) Relative protein quantification of each time point (n=6) for HO-1 (B), Srxn (C), GCLC-1 (D), and CAT-2 (E). Results are expressed as relative protein expression and reported as mean ± SEM. *P<0.05 analyzed by one-way ANOVA followed by Dunnett post-hoc test. Figure S3. The temporal expression of Nrf2 target genes at protein level in the hippocampus after SE. A) The expression of Nrf2 target genes, HO-1, Srxn, GCLC-1, and CAT-2 in the cortex following kainic acid induced-SE at the protein level using western blot analysis. B-E) Relative protein quantification of each time point (n=6) for HO-1 (B), Srxn (C), GCLC-1 (D), and CAT-2 (E). Results are expressed as relative protein expression and reported as mean ± SEM. *P<0.05 analyzed by one-way ANOVA followed by Dunnett post-hoc test. Figure S4. Male and female associated expression of Nrf2 and target genes in the cortex after SE. Comparison of male and female expression of mRNA levels of Nrf2 (A), and its target genes NQO1 (B), HO-1 (C), Srxn (D), GCLC-1 (E), and CAT-2 (F) in the cortex at different time points after kainic acid-induced status epilepticus (SE) (Male (n=3)/Female (n=3) rats for each time point). Results are expressed as relative expressio [file 13578_2022_951_MOESM1_ESM.docx]

**Nrf2 is predominantly expressed in hippocampal neurons in a rat model of temporal lobe epilepsy**

Sereen Sandouka^1^, Aseel Saadi^1^, Prince Kumar Singh^1^, Rhoda Olowe^1^, Tawfeeq Shekh-Ahmad^1^*

^1^The Institute for Drug Research, The School of Pharmacy, Faculty of Medicine, The Hebrew University of Jerusalem, Jerusalem, Israel – 91120.

*Correspondence: Tawfeeq Shekh-Ahmad, The Institute for Drug Research, The School of Pharmacy, Faculty of Medicine, The Hebrew University of Jerusalem, Jerusalem, Israel – 91120. E-mail: [Tawfeeq.Shekh-Ahmad@mail.huji.ac.il](mailto:Tawfeeq.Shekh-Ahmad@mail.huji.ac.il)


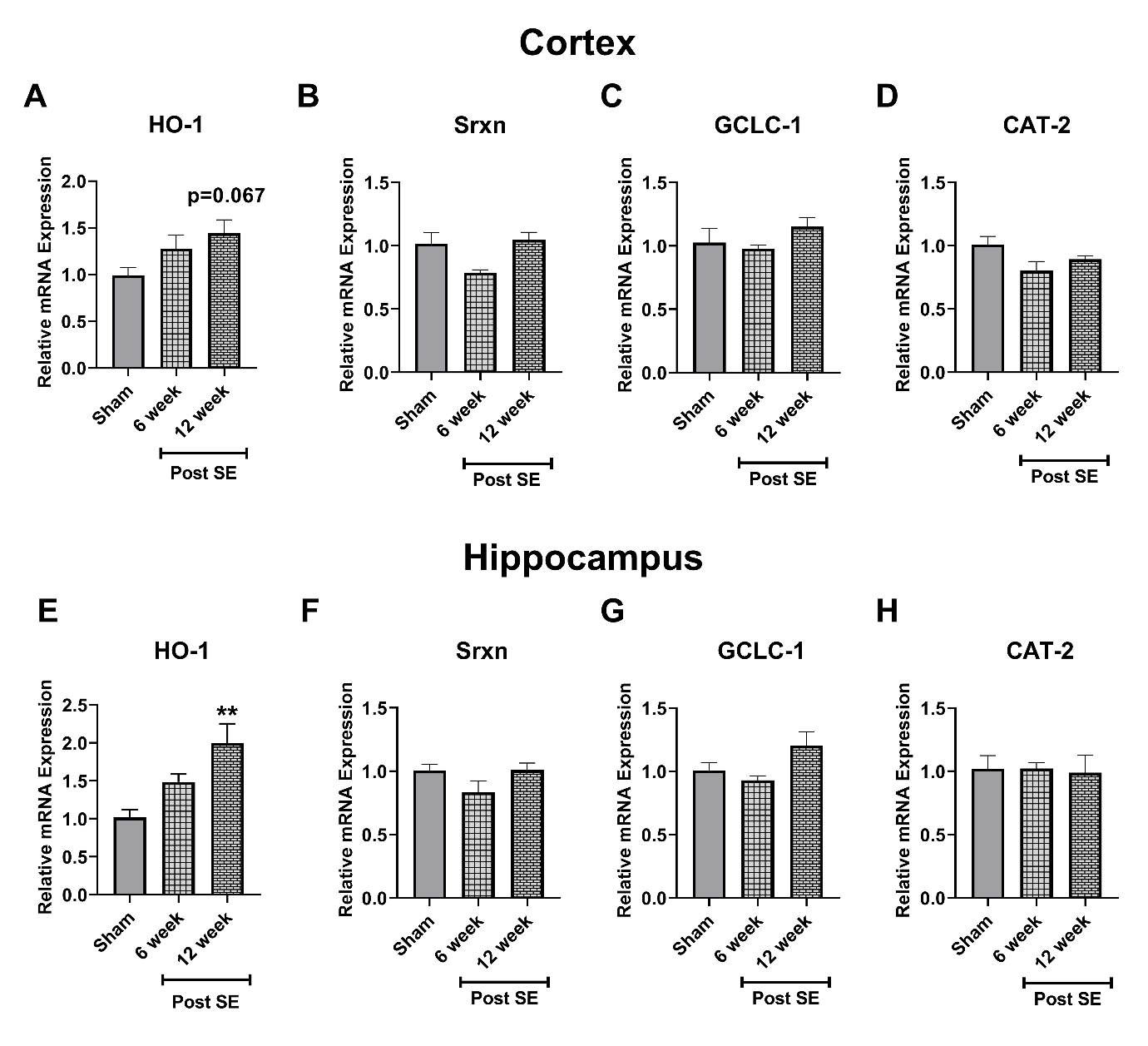


**Additional file 1: Figure S1. Temporal mRNA expression of Nrf2 downstream target genes during late epileptic phase:** (A) heme oxygenase-1 [HO-1], (B) Sulfiredoxin [Srxn], (C) Glutamate-Cysteine Ligase Catalytic Subunit 1 [GCLC1], and (D) Catalase [CAT-2] in the cortex (A-D) and the hippocampus (E-H) following kainic acid induced-SE (n=6 rats for each time point). Results are expressed as relative mRNA expression and reported as mean ± SEM. ***P<0.01 analyzed by one-way ANOVA followed by Tukey’s post-hoc test.*


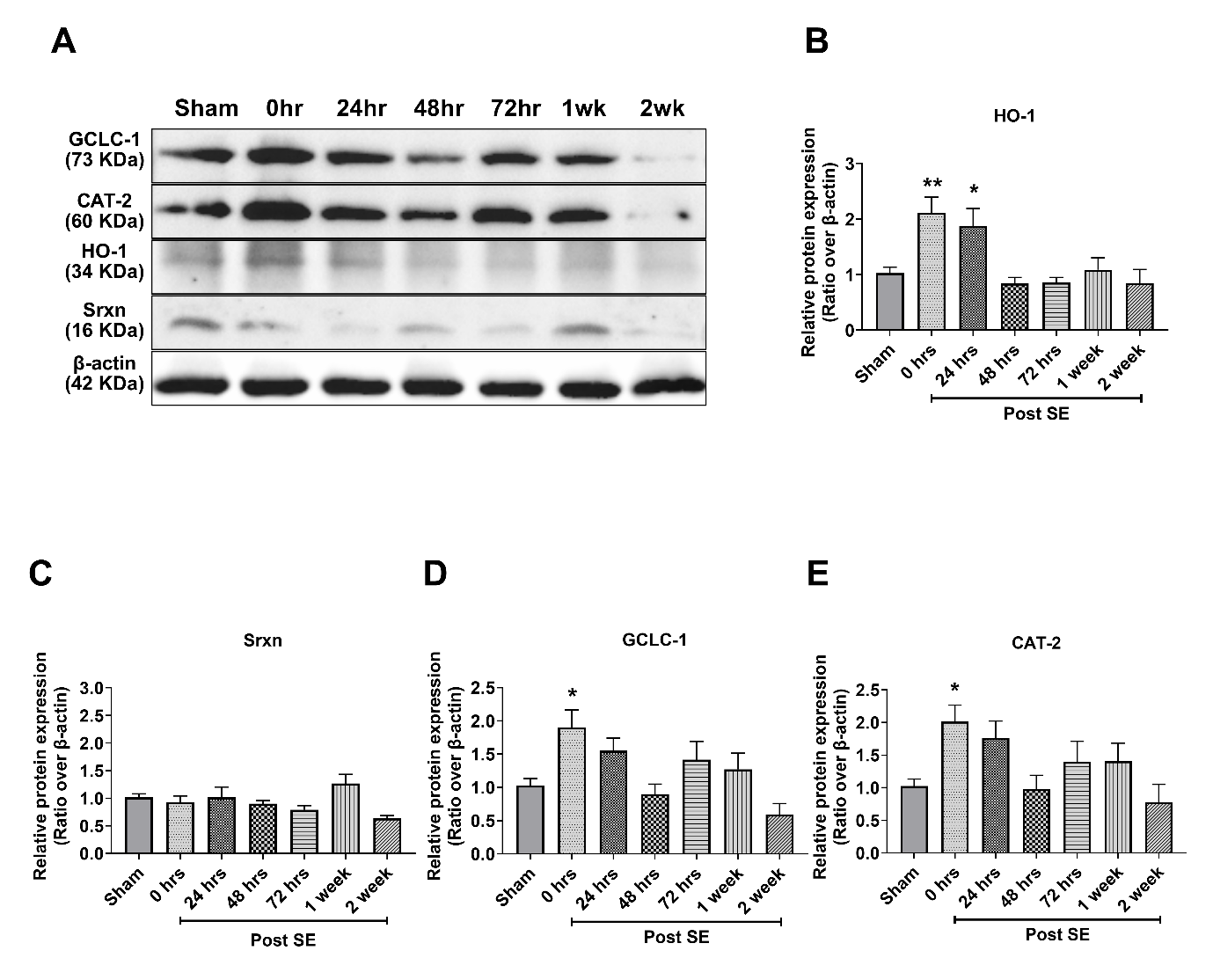


**Additional Figure S2. The temporal expression of Nrf2 target genes at protein level in the cortex after SE.**

A) The expression of Nrf2 target genes, HO-1, Srxn, GCLC-1, and CAT-2 in the cortex following kainic acid induced-SE at the protein level using western blot analysis. B-E) Relative protein quantification of each time point (n=6) for HO-1 (B), Srxn (C), GCLC-1 (D), and CAT-2 (E). Results are expressed as relative protein expression and reported as mean ± SEM. **P<0.05 analyzed by one-way ANOVA followed by Dunnett*post-hoc*test.*


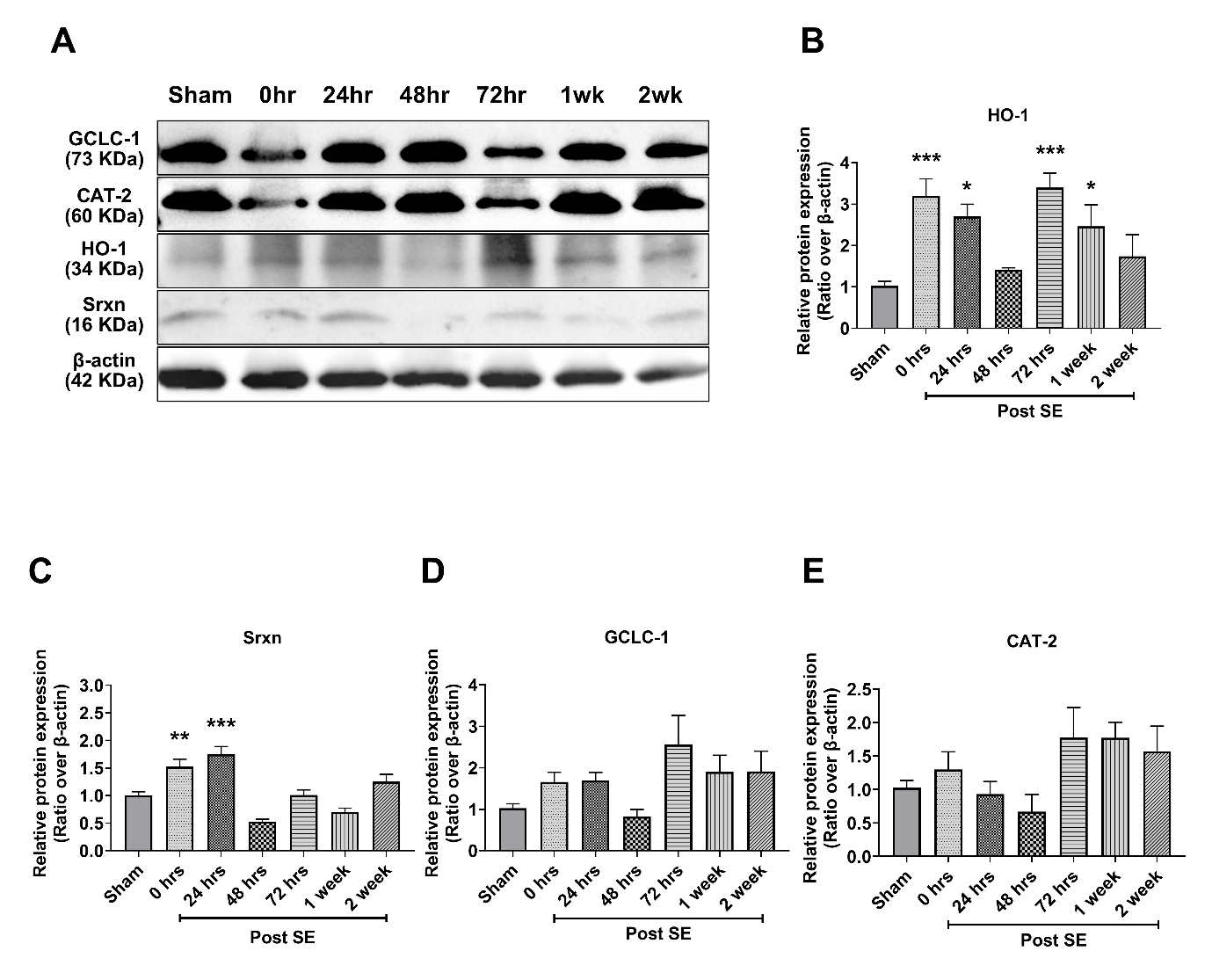


**Additional file 1: Figure S3. The temporal expression of Nrf2 target genes at protein level in the hippocampus after SE.**

A) The expression of Nrf2 target genes, HO-1, Srxn, GCLC-1, and CAT-2 in the cortex following kainic acid induced-SE at the protein level using western blot analysis. B-E) Relative protein quantification of each time point (n=6) for HO-1 (B), Srxn (C), GCLC-1 (D), and CAT-2 (E). Results are expressed as relative protein expression and reported as mean ± SEM. **P<0.05 analyzed by one-way ANOVA followed by Dunnett*post-hoc*test.*


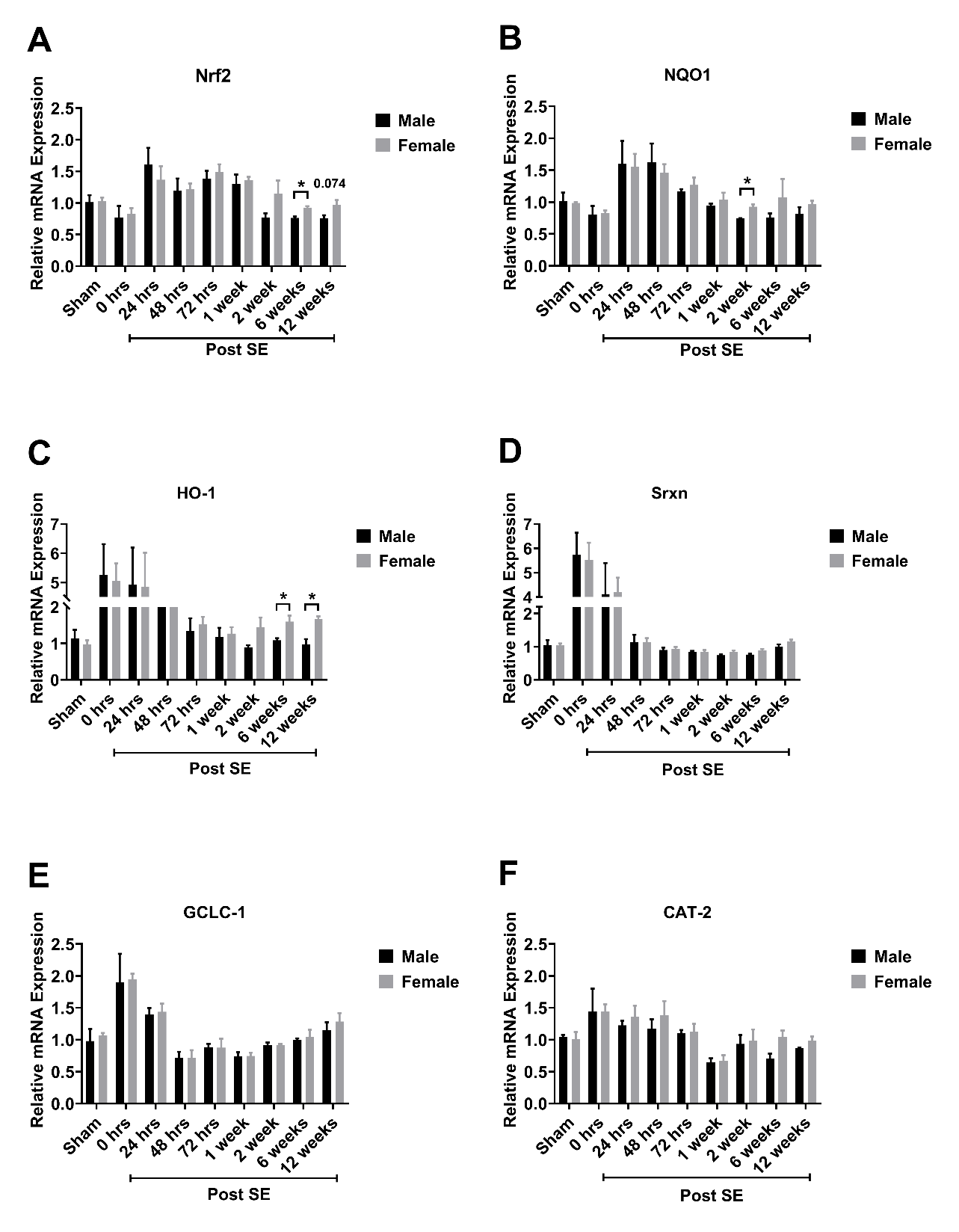


**Additional file 1: Figure S4 Male and female associated expression of Nrf2 and target genes in the cortex after SE.**

Comparison of male and female expression of mRNA levels of Nrf2 (A), and its target genes NQO1 (B), HO-1 (C), Srxn (D), GCLC-1 (E), and CAT-2 (F) in the cortex at different time points after kainic acid-induced status epilepticus (SE) (Male (n=3)/Female (n=3) rats for each time point). Results are expressed as relative expression of mRNA levels and reported as mean ± SEM. **P<0.05 analyzed by unpaired Student’s t-test.*


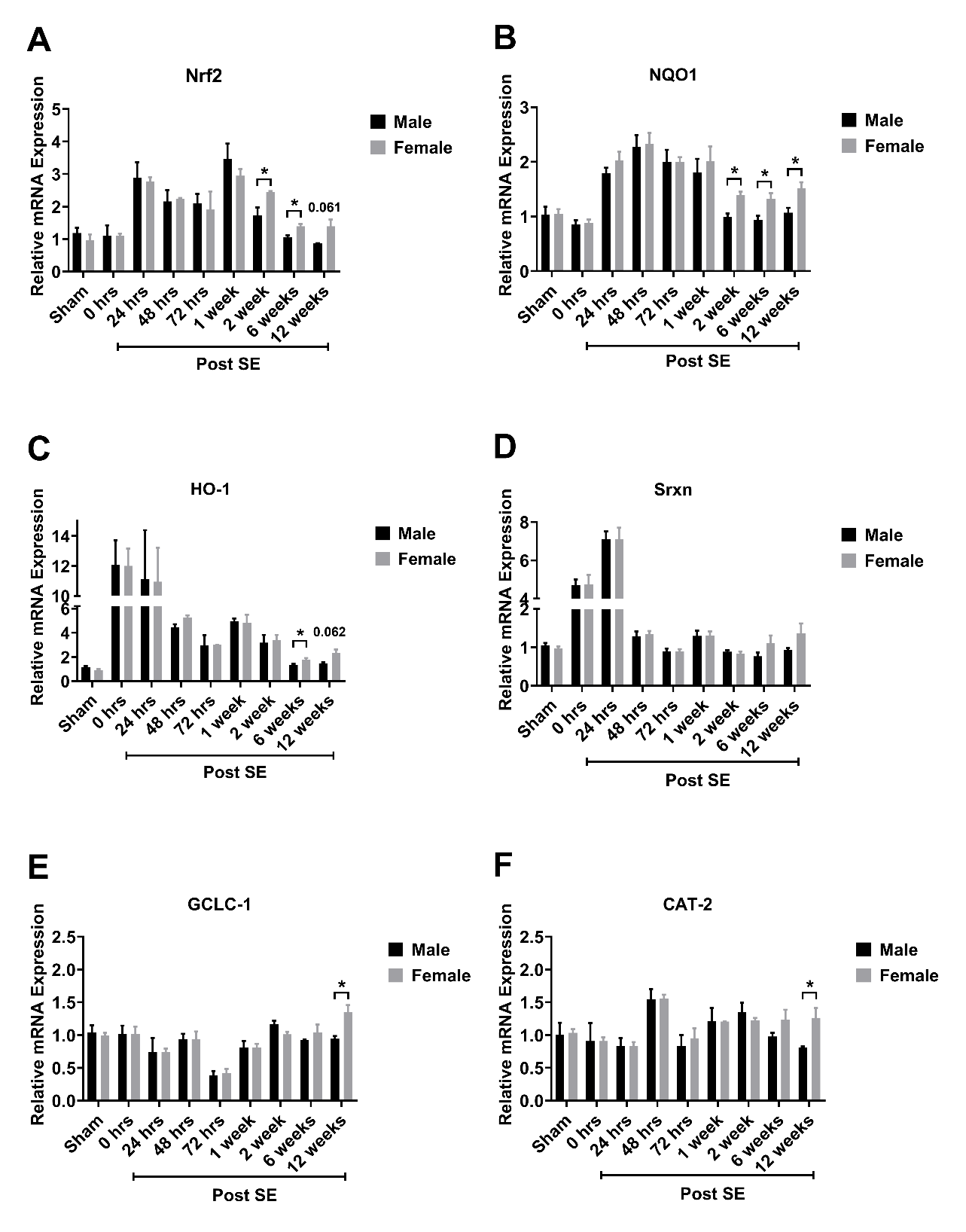


**Aditional file 1: Figure s5. Male and female associated expression of Nrf2 and target genes in the hippocampus after SE.**

Comparison of male and female expression of mRNA levels of Nrf2 (A), and its target genes NQO1 (B), HO-1 (C), Srxn (D), GCLC-1 (E), and CAT-2 (F) in the hippocampus at different time points after kainic acid-induced status epilepticus (SE) (Male (n=3)/Female (n=3) rats for each time point). Results are expressed as relative expression of mRNA levels and reported as mean ± SEM. **P<0.05 analyzed by unpaired Student’s t-test.*


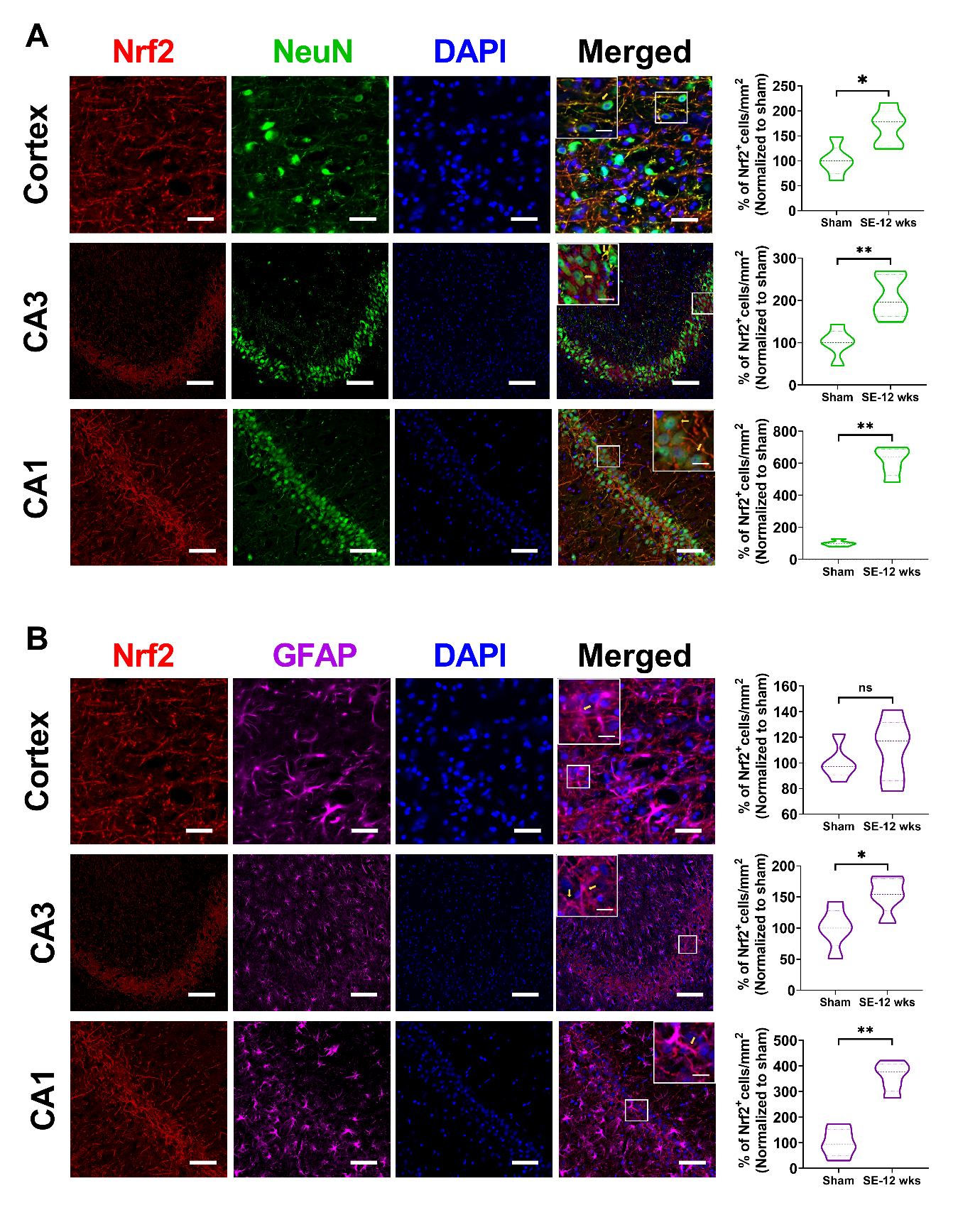


**Additional file 1: Figure S6 Nrf2 expression in neurons and astrocytes in the cortex and hippocampus at 12-weeks after SE.**

A) Representative fluorescent images of rat cortex, CA3, and CA1 regions of the hippocampus (left panels) from animals after 12 weeks of kainic acid induced-SE, illustrating Nrf2 (red), NeuN (green), and DAPI (blue). Scale bar = 50 µm and 15 µm (zoomed view). The right panels represent bar charts summarizing the corresponding quantification of the number of cells double-positive for NeuN and Nrf2 in 1 mm^2^ of tissue, and normalized to the sham group. N=5 rats/group. Results were expressed as mean ± SEM. Cortex: p=0.0317, CA3: p=0.0079, CA1: p=0.0079 compared to sham group by Mann–Whitney U test.

B) Representative fluorescent images of rat cortex, CA3, and CA1 regions of the hippocampus (left panels) from animals after 12 weeks of kainic acid induced-SE, illustrating Nrf2 (red), GFAP (magenta), and DAPI (blue). Scale bar = 50 µm and 15 µm (zoomed view). The right panels represent bar charts summarizing the corresponding quantification of the number of cells double-positive for NeuN and Nrf2 in 1 mm^2^ of tissue, and normalized to the sham group. N=5 rats/group. Results were expressed as mean ± SEM. Cortex: p=0.8413, CA3: p=0.0317, CA1: p=0.0079 compared to sham group by Mann–Whitney U test.
